# Endothelial Dysfunction in Diabetic Retinopathy

**DOI:** 10.3389/fendo.2020.00591

**Published:** 2020-09-04

**Authors:** Fu Gui, Zhipeng You, Shuhua Fu, Hongxi Wu, Yulan Zhang

**Affiliations:** Department of Ophthalmology, The Second Affiliated Hospital of Nanchang University, Nanchang, China

**Keywords:** diabetes, diabetic retinopathy, endothelial cells, endothelial dysfunction, signaling pathways

## Abstract

Diabetic retinopathy (DR) is a diabetic complication which affects retinal function and results in severe loss of vision and relevant retinal diseases. Retinal vascular dysfunction caused by multifactors, such as advanced glycosylation end products and receptors, pro-inflammatory cytokines and chemokines, proliferator-activated receptor-γ disruption, growth factors, oxidative stress, and microRNA. These factors promote retinal endothelial dysfunction, which results in the development of DR. In this review, we summarize the contributors in the pathophysiology of DR for a better understanding of the molecular and cellular mechanism in the development of DR with a special emphasis on retinal endothelial dysfunction.

## Introduction

Diabetic retinopathy (DR) is one of the major complications of diabetes. In 2019, there were about 463 million adults with diabetes worldwide according to the International Diabetes Federation. Diabetes has been one of the most common causes for death in adults aged 20–74 years old (1). DR is resulted from long-term accumulated damages by hyperglycemia or other factors (such as hypertension) to the microvessels in the retina (2). It is a major cause of blindness and other relevant retinal diseases (such as diabetic macular edema and DME) and has received particular attention (3).

Although diagnosis and treatment at the early stage can reduce vision loss in some patients, DR remains a serious threat to vision and patients' quality of life. DR and relevant retinal diseases are related to retinal vascular dysfunction. Although DR now is more precisely defined as a neurovascular disease rather than a microvascular disease (4), retinal microvasculopathy remains the main pathological change of DR. Hyperglycemia causes retinal microvasculopathy, inflammation, and retinal neurodegeneration, all of which result in the breakdown of the blood–retinal barrier (BRB) and damages the endothelium to form acellular capillaries and edema in retinal vascular structure (5).

Diabetic retinopathy has two stages: non-proliferative diabetic retinopathy (NPDR) and proliferative diabetic retinopathy (PDR). NPDR is an early stage of DR which is characterized by loss of pericytes from retinal capillaries to form acellular capillaries, increase vascular permeability, and break down the inner endothelial BRB (6). It is usually asymptomatic. PDR is an advanced stage at which new, vulnerable, and tortuous blood vessels are formed in the retina. They can cause fibrovascular epiretinal membranes, vitreous hemorrhage, and retinal detachment, all of which contribute to vision loss (6).

The underlying molecular mechanisms associated with vascular dysfunction, especially endothelial dysfunction, in DR are multifactorial. Extensive studies have been performed to identify factors that are associated with endothelial dysfunction in DR, such as advanced glycosylation end products (AGEs) and receptors (RAGE), disruption of peroxisome proliferator-activated receptor-γ (PPARγ), chronic inflammation, leukotasis (7–10), oxidative stress, and dysregulated growth factors, cytokines, and microRNA (miRNA) networks (10–13). Here, we review the available data and summarize on AGE, PPARγ, inflammation, miRNA, and signaling pathways that contribute to endothelial dysfunction in the development of retinal microvasculopathy and analyze the challenges in understanding the pathology of DR.

## Advanced Glycosylation End Products and Receptors in Endothelial Dysfunction of DR

AGEs are glycated proteins or lipids which are resulted from exposure to hyperglycemia over time. Hyperglycemia causes the activation of the polyol pathway to produce fructose, fructose-3-phosphate, and 3-deoxyglucosone, which are glycosylating agents (14). Glucose and the increased glycosylating agents form covalent bonds with the proteins or lipids to form AGEs.

**Figure 1:**
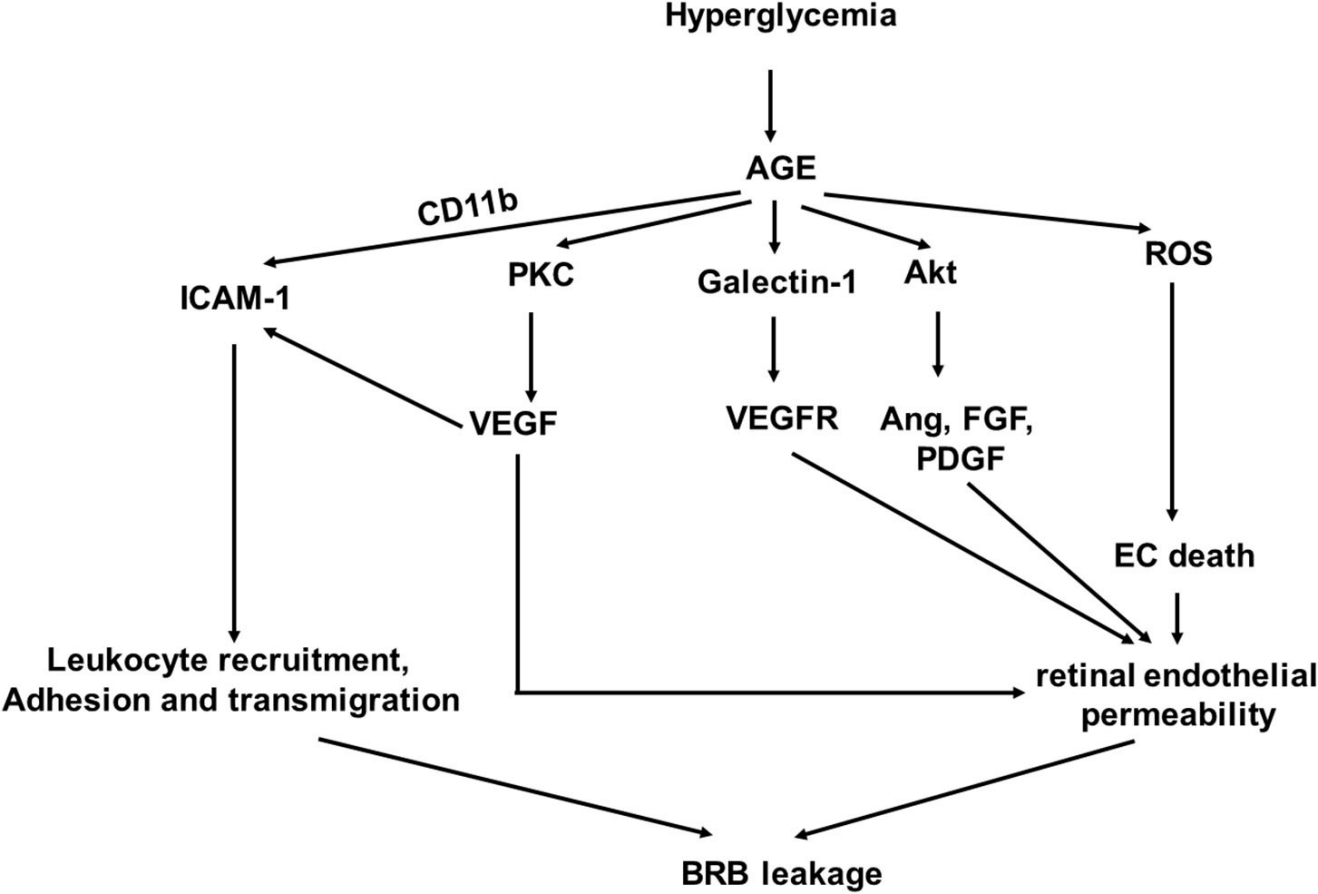
A schematic model of interaction networks mediated by glycosylation end products (AGE) that contributes to blood retinal (BRB) leakage in diabetic retinopathy.

AGEs are detrimental to vascular cells and have been shown to promote the development and progression of DR (Figure 1) (15, 16). A single dietary AGE can acutely impair endothelial function in diabetic and non-diabetic subjects (17). AGE accumulation in cells is a result of their generation from glucose-derived dicarbonyl precursors through non-enzymatic glycation reaction, which is called the “Maillard reaction” (18). Intracellular AGEs interfere with cell function by disrupting molecular conformation, altering enzyme activity, reducing degradation ability, and inhibiting receptor recognition (19).

Studies have shown that accumulation of AGEs in the retinal blood vessel walls is detrimental (10, 20, 21). It causes increased permeability of retinal endothelial cells (ECs) to induce vascular leakage (20). AGEs can upregulate AGE receptor (RAGE) gene expression levels in pericytes and microvascular ECs (21). Activation of RAGEs transduces multiple signals, leading to increased oxidative stress and synthesis of growth factors, adhesion molecules, and pro-inflammatory cytokines (22–24) and resulting in activation of nuclear transcription factors, such as NF-κB (25, 26).

The interaction of AGEs and RAGE increases reactive oxygen species (ROS) product in ECs (22, 27, 28). Both nicotinamide adenine dinucleotide phosphate (NAPDH) oxidase and the mitochondrial electron transport system are involved in ROS generation by AGE signal transduction in ECs (29) as the inhibition of both significantly reduced AGE-induced ROS production (29). Hyperglycemia-induced mitochondrial superoxide can be abrogated by inhibition of AGE-RAGE-mediated mitochondrial permeability transition *in vitro* (30). Similarly, lowering AGEs with alagebrium reduced mitochondrial superoxide generation. The AGE-mediated ROS generation is at least partly through NF-κB activation and subsequent TNF-α production in ECs (31).

The interaction of AGEs and RAGE also promotes expressions of growth factors, proinflammatory cytokines and chemokines, and adhesion molecules through the mitogen-activated protein kinase (MAPK) pathway, leading to NADPH oxidase-mediated ROS generation and translocation of NF-κB (23, 32).

AGEs upregulate VEGF expression in retinal ECs (33). VEGF expression and PKC activation induced by AGEs in retinal ECs were inhibited by the PKC inhibitor and the antioxidant drug and compounds, but not compound that did not have antioxidant property. VEGF is known to stimulate angiogenesis and neovascularization, which are involved in the pathogenesis of proliferative retinopathy (15). The levels of VEGF in ocular fluid are associated with the breakdown of the BRB, which increases microvascular permeability (34).

In addition to VEGF, other angiogenic factors, including angiopoietin-1 (Ang-1) and angiopoietin-2 (Ang-2), fibroblast growth factor (FGF), and platelet derived growth factor (PDGF), have been shown to be upregulated in retinal capillary ECs through Akt-mediated signaling activated by AGEs (15). AGEs can stimulate basic FGF expression in cultured Müller cells to affect pathogenesis of DR (35).

Endothelial cell-expressed RAGE can act as Mac-1 (CD11b) ligand and work cooperatively with Intercellular Adhesion Molecule-1 (ICAM-1) to mediate leukocyte adhesion during the acute inflammation *in vivo* (36). VEGF induces ICAM-1 expression on retinal ECs to promote monocyte adhesion (37). Increased ICAM-1 expression in the retinal ECs contributes to microvascular leukostasis, the adhesion, and transmigration of leukocytes to endothelium, in DR (38, 39). AGE induces specific galectin-1 expression, which may be correlated with disease activity in DR as galectin-1 can bind to VEGF receptors-1 and−2 in ECs, resulting in angiogenesis and vascular permeability, respectively (40, 41).

AGE upregulates PKC activation, increases ROS production, and promotes synthesis of growth factors, adhesion molecules, and pro-inflammatory cytokines. Understanding the underlying cellular and molecular pathogenesis mechanism of AGE-induced endothelial dysfunction in DR will facilitate early detection of DR and identify novel anti-AGE drugs, which can block the biological activity of AGEs.

## Disruption of PPARγ in Endothelial Dysfunction of DR

**Figure 2:**
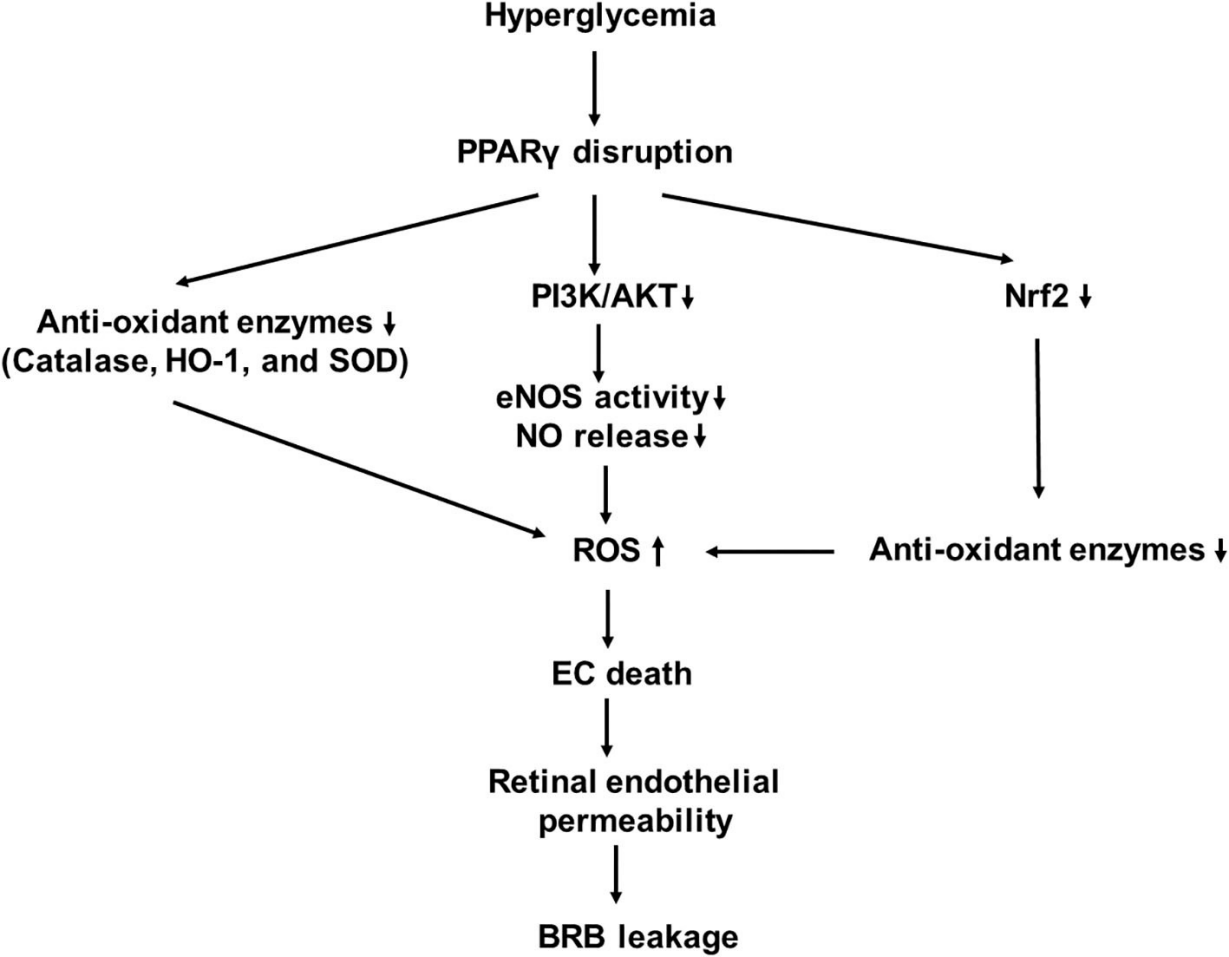
A schematic model of interaction networks mediated by proliferator-activated receptor-γ (PPARγ) disruption that contributes to blood retinal (BRB) leakage in diabetic retinopathy.

PPARγ is a nutrient sensor that controls a variety of homeostatic functions. Its disruption leads to disorders of fatty acid/lipid metabolism, insulin resistance, and vascular pathology. Endothelial PPARγ is essential for preventing endothelial dysfunction with aging (42, 43). Impaired endothelial PPARγ causes age-related vascular dysfunction. PPARγ activation mediates antioxidant response and nitric oxide (NO) product in ECs. It induces increased expression of nuclear factor of kappa light polypeptide gene enhancer in B-cell inhibitor (IκB), phosphatase and tensin homolog (PTEN), and Sirtuin 1 (SIRT1), all of which interfere with the activation of NF-κB (44). PPARγ promotes the expression of antioxidant enzymes, including catalase, heme oxygenase-1 (HO-1), and superoxide dismutase (SOD), which lead to a reduction of the ROS product (44). PPARγ inhibits diabetes-induced retinal leukostasis and microvascular leakage through its role on increasing expression of endothelial nitric oxide synthase (eNOS) activity, reducing oxidative stress, inhibiting apoptosis, inflammation, and angiogenesis (43). PPARγ receptors have been shown to be downregulated in the diabetic eye, and their disruption is involved in the pathogenesis of DR (Figure 2) (45, 46).

### Endothelial Nitric Oxide Synthase and Nitric Oxide

Nitric oxide produced by eNOS is a major medium which mediates relaxation and vasodilatation of the vessels. Production and bioavailability of NO are reduced in the early stages of DR (47), while PPARγ activation increases production and bioavailability of NO. PPARγ ligands, such as 15-deoxy-Δ (12, 14)-prostaglandin J_2_ (15d-PGJ_2_), rosiglitazone, and nitrooleate, are able to increase eNOS activity and NO release through increased interaction between heat shock protein 90 (HSP90) and eNOS (48, 49). Rudnicki et al. assessed the effect of 3 thiazolidinediones (TZDs), GQ-32, GQ-169, and LYSO-7, on NO, ROS, and adhesion molecules on ECs (50). Although all of three activated PPARγ and enhanced the intracellular NO level, only LYSO-7 significantly increased the NO release from ECs. They all suppressed the adhesion molecule expressions induced by TNF-α. Additionally, GQ-169 and LYSO-7 inhibited ROS production in response to high glucose. PPARγ activation decreases expressions of NADPH oxidase subunits and enhances the expression of superoxide dismutase (SOD), which result in enhanced NO bioavailability to reduce oxidative stress in the membrane of human umbilical vein endothelial cells (HUVECs) (51).

Aleglitazar, a dual-PPARα/γ agonist, has been shown to increase eNOS, Akt, and telomerase activities in circulating angiogenic cells (52). Rosiglitazone increases eNOS and Akt activity and NO synthesized by endothelial progenitor cells (EPCs), which are reduced by AGEs. Its beneficial effect can be blocked by the eNOS inhibitor and phosphoinositide 3-kinase/protein kinase B (PI3K/AKT) inhibitor, indicating that rosiglitazone can improve AGE-induced EPC dysfunction by AGEs through upregulating the AKT/eNOS signal pathways in EPCs (53).

### Oxidative Stress

Hyperglycemia induces oxidative stress in patients with diabetes. Oxidative stress, which resulted from increased NADPH oxidase, is a key factor involved in the development of DR (11, 12). It activates inhibitory redox-regulated transcription factors to attenuate PPARγ expression and activity in vascular ECs (54). PPARγ exerts its antioxidative function through transcriptional activation of a number of antioxidant genes (55–57). The major ROS produced in response to hyperglycemia is superoxide anion (${\text{O}}_{2}^{-}$) which combines with NO to produce peroxynitrite (ONOO?). This leads to decrease in NO bioavailability, which causes endothelial dysfunction (58).

PPARγ can transcriptionally regulate HO-1 expression in vascular cells (56). Its activation induces the expression of glutathione peroxidase 3 (GPx3), which detoxifies the extracellular H_2_O_2_ level. The inhibition of GPx3 expression prevents the antioxidant effects of the PPARγ ligand on oxidative stress in insulin-resistant cells (59). Troglitazone and pioglitazone increases Cu^2+^, Zn^2+^-superoxide dismutase (CuZn-SOD) gene expression and protein levels (60). 15d-PGJ_2_ or ciglitazone reduces gene and protein expressions of the NADPH oxidase subunits, such as nox-2 and nox-4, and stimulates protein expression and activity of Cu/Zn-SOD in HUVECs (51).

Oxidative stress also impairs reendothelialization ability of EPCs derived from patients with diabetes, while rosiglitazone improves reendothelialization EPC therapy potential by reduces NADPH oxidase activity (61). Pioglitazone can inhibit NADPH oxidase p22 (phox) and Rac1. The latter is responsible for producing ROS, which negatively regulates EPC migration, proliferation, and differentiation. Recently, Liu et al. have demonstrated that PPARγ activation can transcriptionally upregulate the expression of long intergenic noncoding RNA 01230 (Linc01230), which reduces oxide low-density lipoprotein-induced endothelial dysfunction and affects the phosphorylation of AKT (62).

In addition to directly modulating oxidative stress response, PPARγ can indirectly modulate through nuclear factor E2-related factor 2 (Nrf2) activation (63). Nrf2 is a transcription factor that regulates the expression of antioxidant proteins (64, 65). When transported inside the nucleus, Nrf2 works with other activators to form a protein complex. The latter binds to the antioxidant response elements (AREs) in the upstream promoter regions of cytoprotective and detoxifying genes to activate their transcription (64, 66). Studies have shown that there is a reciprocal transcriptional regulation between Nrf2 and PPARγ pathways to enhance the expression of one another (57, 63). PPARγ is upregulated in mice in which Nrf2 is increased and is downregulated in *Nrf2*^−/−^ mice (57, 67). ChIP assays have shown that with cofactor Brg1, Nrf2 is coimmunoprecipitated on the ARE containing the upstream promotor region of PPAR-γ (67). Nrf2 expression is reduced in mice with decreased PPARγ (68). PPARγ may act directly or through the upstream pathway to activate Nrf2 (57). A peroxisome proliferator response element, through which PPARγ regulates Nrf2 expression, in the promoter region of Nrf2 gene has been proposed (57). Future studies are needed to prove a direct effect of PPARγ on Nrf2.

Although PPARγ activation promotes antioxidant response and promotes the expression of antioxidant enzymes and NO product in ECs, PPARγ receptors are downregulated in the diabetic eye and their suppression is involved in the pathogenesis of DR (45, 46). Thus, it is not easy to fully reverse endothelial dysfunction using only PPARγ ligands in DR. Strategies aiming to improve the sensitivity or upregulate PPARγ receptor expression in ECs of DR are valuable therapeutic approaches.

## Inflammation and Endothelial Dysfunction of DR

**Figure 3:**
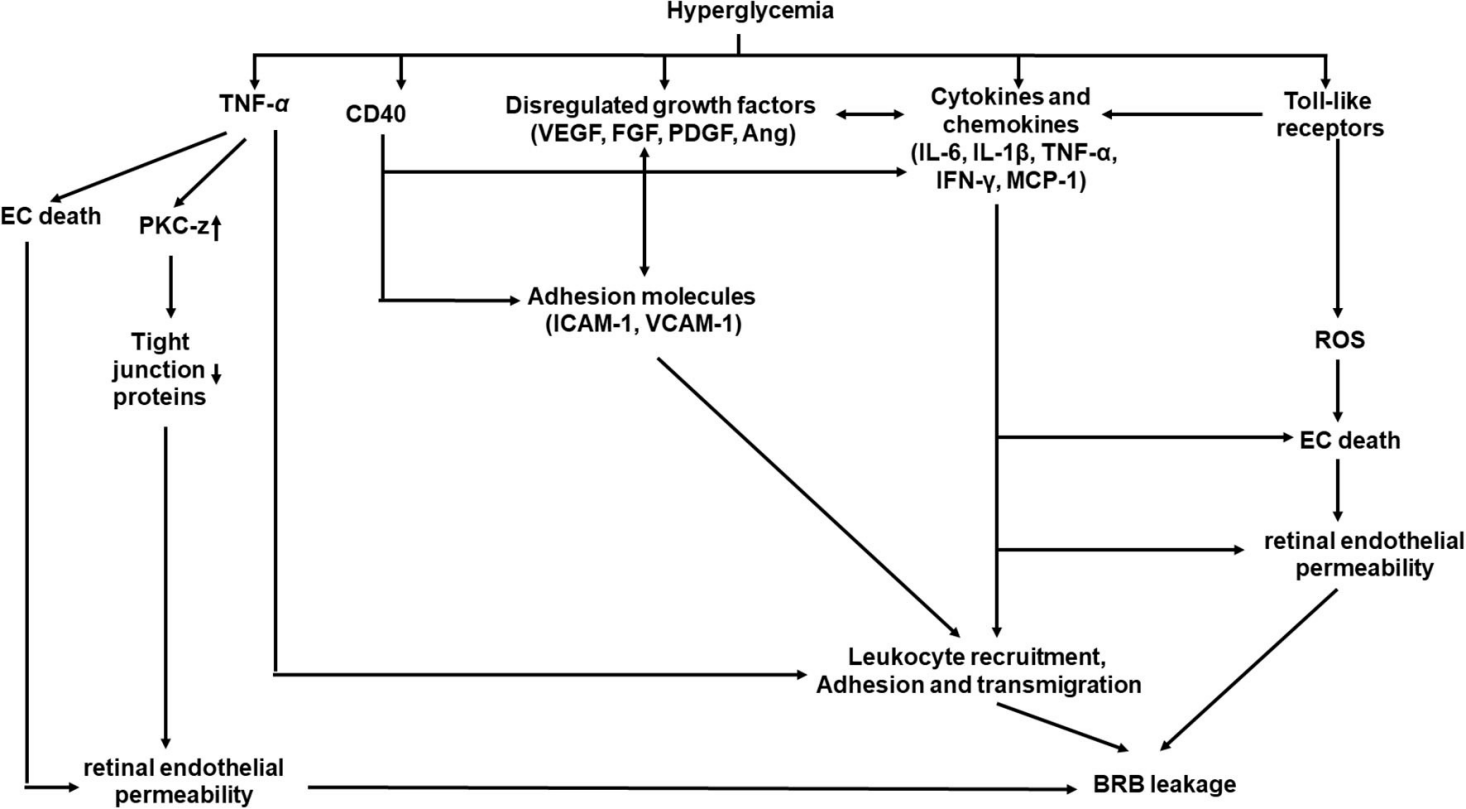
A schematic model of interaction networks mediated by inflammation that contributes to blood retinal barrier (BRB) leakage in diabetic retinopathy.

Inflammation plays important roles in structural and molecular changes associated with DR (Figure 3) (69, 70). Systematically, hyperglycemia causes AGE formation and increases ROS product and plasma proinflammatory cytokines, including TNF-α and interleukin-6 (IL-6) (11, 15, 16, 71). Locally, retinal hypoxia leads to the release of many molecules in the vitreous, including proinflammatory cytokines [TNF-α, interleukin-1β (IL-1β), IL-6, interleukin-8 (IL-8), and interferon-γ (IFN-γ), etc.), chemokines [monocyte chemoattractant protein-1 (MCP-1)], growth factor (VEGF, FGF, and PDGF etc.), adhesion molecules [ICAM-1 and vascular cellular adhesion molecules-1 (VCAM-1)], and receptors (CD40 and Toll-like receptors), from retinal vascular cells, inflammatory cells, and/or glial cells (72, 73).

### Cytokines

Proinflammatory cytokines, such as TNF-α, IL-1, IL-6, IL-8, and IFN-γ, are the major players in inflammation in DR. Increased concentrations of TNF-α, IL-1, IL-6, IL-8, and IFN-γ have been found in the vitreous (74) or in aqueous humor (75) of patients with DR. Their concentrations may be associated with the severity of DR (75).

#### TNF-α

TNF-α can attract inflammatory cells, induce inflammatory cytokine release, and cause necroptosis on targeting cells and proliferation of immune cells (76). TNF-α can be synthesized and released by activated microglia, ECs, macroglia, Müller cells, and neurons (75). Increased levels of TNF-α have been found in PDR. A study of meta-analysis indicated that the level of TNF-α in DR patients was significantly different from that in healthy controls (77). There is a strong correlation between plasma TNF-α levels and the severity of (78). TNF-α concentration has been suggested as a biomarker for the severity of DR as TNF-α in tears increases and is highly correlated with DR severity (79). TNF-α increases retinal EC permeability by reducing the expression of tight-junction proteins through activation of protein kinase C zeta and NF-κB (80). TNF-α is also a chemoattractant for leukocytes to stimulate leukocyte adhesion (78).

TNF-α is critical mediator for later complications in DR. In a TNF-α knockout mouse model, Huang et al. demonstrated that TNF-α is not required for early BRB breakdown in DR (81). However, the absence of TNF-α significantly suppressed BRB breakdown in 6-month-old mice with diabetes. Consistently, apoptosis of ECs, pericytes, and neurons was inhibited in TNF-α knockout mouse models with or without diabetes. However, recent studies showed that a higher level of TNF-α was observed in patient eyes with NPDR than with PDR (75), (82). The discrepancy may indicate the transit of NPDR into PDR.

#### IL-1 β

IL-1 β has been shown to be important in mediating innate immunity and contributing directly to several retinal degenerative diseases, including DR (83). IL-1β can be produced by microglial cells, Müller cells, and astrocytes (9). A significantly high level of IL-1β has been detected in the vitreous humor and serum of patients with PDR (83, 84). The aqueous concentrations of IL-1β from the eye anterior chamber increased with the severity of DR (75). Intravitreal injection of IL-1β caused apoptosis of capillary cells in retinal microvessels and acellular capillaries (85), which are probably mediated by NF-κB and caspase-3 activation (86). IL-1β is cytotoxic to choroidal blood vessels in the choroid, which may lead to the death of the retinal pigment epithelium and damage photoreceptor integrity (87). Hyperglycemia induces Müller cells to produce high levels IL-1β, which induces the expression of pro-death cytokine IL-8 (88). Actually, IL-1β is a more potent inducer of IL-8 expression than TNF-α, IL-6, VEGF, and IFN-γ in Müller cells through the p38MAPK and extracellular signal-regulated protein kinase 1 and 2 (ERK1/2) pathways (88). IL-1β is also a stronger IL-6 inducer than TNF-α, IL-8, VEGF, and IFN-γ in Müller cells through upregulation of the p38MAPK/NF-κB pathway (89). Hyperglycemia triggers retinal ECs to upregulate the expression of IL-1β, which serves as an autocrine or paracrine to stimulate IL-1β expression in ECs or macroglial cells to a sustained overexpression of IL-1β (90). IL-1β leads to ROS release and promotes NF-κB translocation to the nucleus to create a continuous inflammatory response (91).

#### IL-6

IL-6 is a member of the pro-inflammatory cytokines involved in expressing of a variety of proteins in regulating angiogenesis, immune responses, and vascular permeability (92). IL-6 can be produced by microglia and astrocytes (9). Significantly higher intravitreal IL-6 levels are found in patients with NPDR and PDR, (75, 82, 93, 94) and its intravitreal concentration correlates with the severity of PDR (95) and retinal macular thickness (96). Increased intravitreal concentration of IL-6 may be associated with the progression of DR from the NPDR to active PDR (95). The increased IL-6 levels may be independent of hypoxia-inducible factor-1α (HIF-1α) or NF-κB activity in the vitreous of PDR (97). However, animal study suggests that HIF-1α still regulates IL-6 expression in diabetic retina as increased HIF-1α, IL-6, and TNF-α are found in diabetic retina of diabetic rats, which can be decreased by the HIF-1α inhibitor (98). IL-6 promotes leukocyte adhesion, microvascular leakage, and TNF-α product in microglial cells in diabetes as these pathological phenotypes were dramatically reduced in the IL-6 knockout mice with diabetes (10).

#### IL-8

IL-8 is not only a potent angiogenic factor but also a chemoattractant for neutrophils and T lymphocytes (69). It can be produced by Müller glial cells, retinal ECs, and astrocytes. Although IL-8 has been detected both in the vitreous (9, 74) or aqueous humor (75, 99) of DR patients, it is higher in the eyes with NPDR than in the eyes with PDR (82). Elevated vitreous IL-8 level seems to correlate with poorer visual acuity in patients with diabetes, suggesting that IL-8 may cause visual acuity loss as DR progression (100). IL-8 has a strong correlation in vitreous and aqueous of patients with PDR (101). IL-8 is induced in Müller cells in response to IL-1β or TNF-α (88), as well as VEGF in microvascular ECs (102).

#### IFN-γ

IFN-γ is an immunoregulatory cytokine which belongs to the Th-1 group lymphocytes. It signals innate immune system responses by recruiting and activating macrophage and cytotoxic T cells to produce a pro-inflammatory effect (103). Increased IFN-γ was observed in the vitreous or aqueous humor of patients with diabetes or with DR (104, 105). In contrast, increased aqueous IFN-γ was only observed in patients with NPDR or PDR (75). IFN-γ was increased in the retina of rats with diabetes (106). IFN-γ induced migration of microglial cells in the subretinal space to affect the ocular microenvironment in response to inflammation (107). Over-expressing IFN-γ in the retina caused intraocular cellular infiltration, photoreceptor death, corneal clouding, cataract formation, and epithelial and microglial proliferation (108). IFN-γ-increased HUVEC permeability is, at least, partly related to its inhibition on NO production: IFN-γ significantly attenuates basal NO concentration and reduces NO increment in the presence of an NO donor in HUVECs (109). IFN-γ-induced disorganization of endothelial junctional integrity through a mechanism involving Rho-kinase mediated cytoskeletal contractions (110). IFN-γ together with TNF-α and IL-β downregulated the HSP27 expression, which led to apoptosis of retinal capillary ECs (111).

### Chemokine: MCP-1

Monocyte chemoattractant protein-1 attracts and activates monocyte and macrophages (112, 113) and stimulates fibrosis and angiogenesis (114). MCP-1 is produced by Müller cells, microglia cells, astrocytes, retinal neurons, ECs, and retinal pigment epithelial cells in patients with diabetes (115). The migration of monocyte into the retina is mediated by MCP-1 coupling to its receptor CCR2 (116). Elevated MCP-1 has been observed in ocular tissues from patients with NPDR or PDR, (10, 82, 104, 117) and its level is higher in the vitreous than in the serum (74). The vitreous MCP-1 level has been shown to be associated with DR severity (100). Intravitreal increase in MCP-1 level may be associated with the progression of NPDR to active PDR (95). Through increasing vascular cell permeability and leukocytes' recruitment, MCP-1 affects BRB in animal eyes of DR (118). In response to IL-1β or TNF-α, retinal ECs or microglial cells will express a high level of MCP-1 to attract macrophages (119), which may adhere to the retinal capillary endothelium, which leads to capillary occlusion and retinal ischemia (120). TNF-α and IL-6 produced by glial cells and microglial cells can stimulate ECs to release MCP-1, IL-6, and VEGF, all of which increase vascular permeability in NPDR (121). MCP-1 exerts its cytotoxic effect through oxidative stress produced by activated macrophage and microglia (122). Although MCP-1 is a potent inducer of angiogenesis, its angiogenic effect is achieved through induction of VEGF-A (123, 124). A significantly positive correlation has been observed between the MCP-1 and VEGF in PDR (125). Although lower levels of MCP-1 have been reported in the aqueous humor from NPDR and PDR patients (126, 127), the discrepancy may be due to different sample preservation and measurement techniques used.

### Growth Factor: VEGF

Increased vitreous concentrations of the growth factors, such as VEGF, FGF (128), PDGF (129), placental growth factor (PlGF) (130), angiopoietin (131), insulin-like growth factor (IGF-1) (132), and hepatocyte growth factor (HGF), have been reported in patients with PDR. Among these, VEGF has received particular attention and will be summarized in this section.

Over the decades, VEGF has been recognized as a major angiogenic growth factor, which is responsible for pathologic retinal neovascularization in DR (133). VEGF is an angiogenic factor that not only induces new blood vessel sprouting from preexisting vessels but also increases vascular permeability. In addition to ECs, other retina cells, such as retinal pigment epithelial cells, pericytes, Müller cells, astrocytes, and glial cells, are also able to produce VEGF upon activation or stimulated by long-term high glucose (9, 134–139).

Increased VEGF level has been observed in the vitreous humor and in fibrovascular tissues from eyes with PDR (140–142). Serum and vitreous VEGF levels have been found to correlate with glycemic control in patients with diabetes (143). A strong correlation between increased level of intravitreal VEGF and the development of DR has been detected (144, 145). Vitreous level of VEGF may be correlated with retinopathy activity (142, 146, 147). More recently, serum VEGF level in subjects with diabetes has been proposed to be a biomarker of severity of DR as it is associated with the severity of DR (148, 149).

VEGF is a key regulator of ocular angiogenesis and vascular permeability. It is involved in the pathogenesis of a number of complications of DR, such as DME and PDR (150). It has been shown that intraocular injection of VEGF alone produced many features of NPDR and PDR: areas of non-perfusion capillaries, vessel dilation, and tortuosity arterioles with endothelial hyperplasia and microaneurysm formation (151). A positive correlation has been found between the level of serum VEGF and the grade of the external limiting membrane (ELM) disruption, indicating that an increased level of VEGF is associated with DR severity and the grade of the external limiting membrane disruption (149).

VEGF modulates DR-associated inflammatory responses at the early stage of DR (13). It acts as a pro-inflammatory factor to promote the expressions of other proinflammatory cytokines, chemokines, and adhesion molecules, such as TNF-α, IL-1, IL-6, IL-8, IFN-γ, MCP-1, and ICAM-1 (102, 152, 153). Vice versa, activated glial cells, macrophages, and microglia cells will produce TNF-α, IL-6, and MCP-1, which in turn can stimulate VEGF release from retinal ECs (123). Increased activation of NF-κB in NPDR and PDR subjects may be involved in increased upregulation of VEGF (154). VEGF induces MCP-1, IL-8, TNF-α, and ICAM-1 expression in retinal ECs by activating NF-kB pathways (13, 102, 153). Müller glia-specific VEGF deletion resulted in 48% percent reduction in the NF-κB activation in diabetic mouse retina (13). This was associated with 50% reduction of TNFα and ICAM-1 in retina and 75% reduction of leukocyte adhesion in the retinal microvasculature.

VEGF enhances the leukocyte adhesion to vessel walls through increasing ICAM-1 and VCAM-1 expressions on ECs (155). In addition, VEGF may initiate early diabetic retinal leukocyte adhesion in retinal arterioles through upregulated ICAM-1 expression (152, 156). Increased serum VEGF levels stimulate ROS generation, which causes endothelial activation (157).

### Adhesion Molecules

Studies show that adhesion molecules play important roles in pathogenesis of vascular complications (158). Adhesion molecules participate in cell growth, differentiation, formation of cell junction, or cell polarity, as well as activation, circulation, or accumulation of white leukocytes at the inflammatory site (158). They participate in initiating the process of monocyte and lymphocyte adhesion to ECs and mediate their transmigration (158).

Numerous endothelial molecules, primarily located at junctions, such as ICAM-1, VCAM-1, platelet/endothelial-cell adhesion molecule-1 (PECAM-1), and endothelial leukocyte adhesion molecule-1 (ELAM-1), are involved in leukocyte transmigration (158–160). ICAM-1 and VCAM-1 are upregulated in the conjunctiva of patients with DR (161). Blood serum soluble forms of VCAM-1, ICAM-1, and ELAM-1 are increased in patients with DR (162). ICAM-1 can act cooperatively with RAGE to mediate leukocyte recruitment during acute inflammation *in vivo* (36). The lymphocyte function-associated antigen-1 can distribute to form a ring-like structure to cocluster with endothelial ICAM-1 to mediate the neutrophil transmigration (130). A positive correlation is found between the level of serum ICAM-1 and the grade of the retinal ELM disruption (149), suggesting, in addition to VEGF, that serum ICAM-1 level is also associated with an increased DR severity and the grade of the external limiting membrane disruption (149). Thus, monitoring serum-soluble VCAM-1 levels in patients with diabetes may be clinically useful for assessing the severity and possibly the activity of diabetic retinopathy (163). The soluble ICAM-1 is a biomarker of endothelial injury and inflammation. ICAM-1, VCAM, and ELAM-1 expressions in ECs can be stimulated by IL-1, TNF-α, and VEGF through activation of the NF-κB pathway (152, 164, 165).

PECAM-1 is important in maintaining vascular integrity (166). Although endothelial PECAM-1 homophilic interactions are required for the maintenance of EC barrier function (166), PECAM-1 also promotes leukocyte transmigration by undergoing homophilic interactions with PECAM-1 on monocytes to facilitate transmigration (167). More recently, soluble vascular adhesion protein-1/semicarbazide-sensitive amine oxidase has been shown to generate H_2_O_2_ and toxic aldehyde acrolein, which cause oxidative stress in eyes with PDR (168).

### Receptors

CD40 and Toll-like receptors play roles in inflammation in the development of DR (169–172).

#### CD40

CD40, a member of the TNF receptor superfamily, is expressed not only on monocytes, dendritic cells, and ECs but also on Müller glia, microglial cells, and retinal pigment epithelial cells (169, 170). CD40 expression level is low at basal condition, and its upregulation results in downstream inflammatory response in diseases (173). Plasma-soluble CD40 ligand level is found to be positively associated with DR severity (173). Increased expression of CD40 was found in retinal Müller cells, ECs, and microglial cells of diabetic animals (174). CD40 upregulates ICAM-1, MCP-1, and VEGF expressions in ECs and Müller cells through TNF receptor-associated factors (175). CD40 knockout mice are protected from the development of DR: reduced retinal leukostasis, inhibited capillary degeneration, and diminished ICAM-1 upregulation (174). Diabetes upregulates P2X7 in the retina through CD40 to make retinal ECs susceptible to ATP/P2X7-mediated apoptosis (176).

#### Toll-Like Receptors

TLRs play an important role in innate immune responses and inflammation (177). TLRs promote proinflammatory cytokine expression, which in turn activate TLRs in immune cells to induce EC damage by the ROS product (171, 172). A high level of mobility group protein-1, a ligand of toll-like receptor (TLR)-4, has been found higher in active PDR than in inactive PDR (178). The agonist of TLR-3 can induce the retinal pigment epithelium to secrete MCP-1, IL-8, and ICAM-1 (179). High glucose significantly upregulates TLR-2 and TLR-4 expression and activates NF-kB and increases expression of IL-1β, IL-8, TNF-α, MCP-1, ICAM-1, VCAM-1, and adhesion of monocyte in human microvascular retinal ECs (180). TLR-4 or TLR-2 inhibitor and antioxidant treatment reduces the expressions of TLR-2 and TLR4 and associated downstream inflammatory markers. These suggest that activation of TLR-2 and TLR-4 and downstream signaling are involved in increased inflammation and ROS in DR. Furthermore, retinal photoreceptors are susceptible to mitochondrial oxidative stress and mitochondrial DNA damage in TLR4-mediated innate immune response, leading to visual impairment (181).

Although there is increasing evidence showing that inflammation is a critical contributor to the development of DR, some studies have also demonstrated that DR is not exclusively due to inflammation (182, 183). Thus, the exact underlying molecular mechanisms of inflammation in DR are not yet fully understood. In addition, inflammation is a complex cascade; thus, therapeutics targeting at one factor may be insufficient. Drugs that inhibit multiple factors in inflammation may help to control DR.

## miRNAs

**Figure 4:**
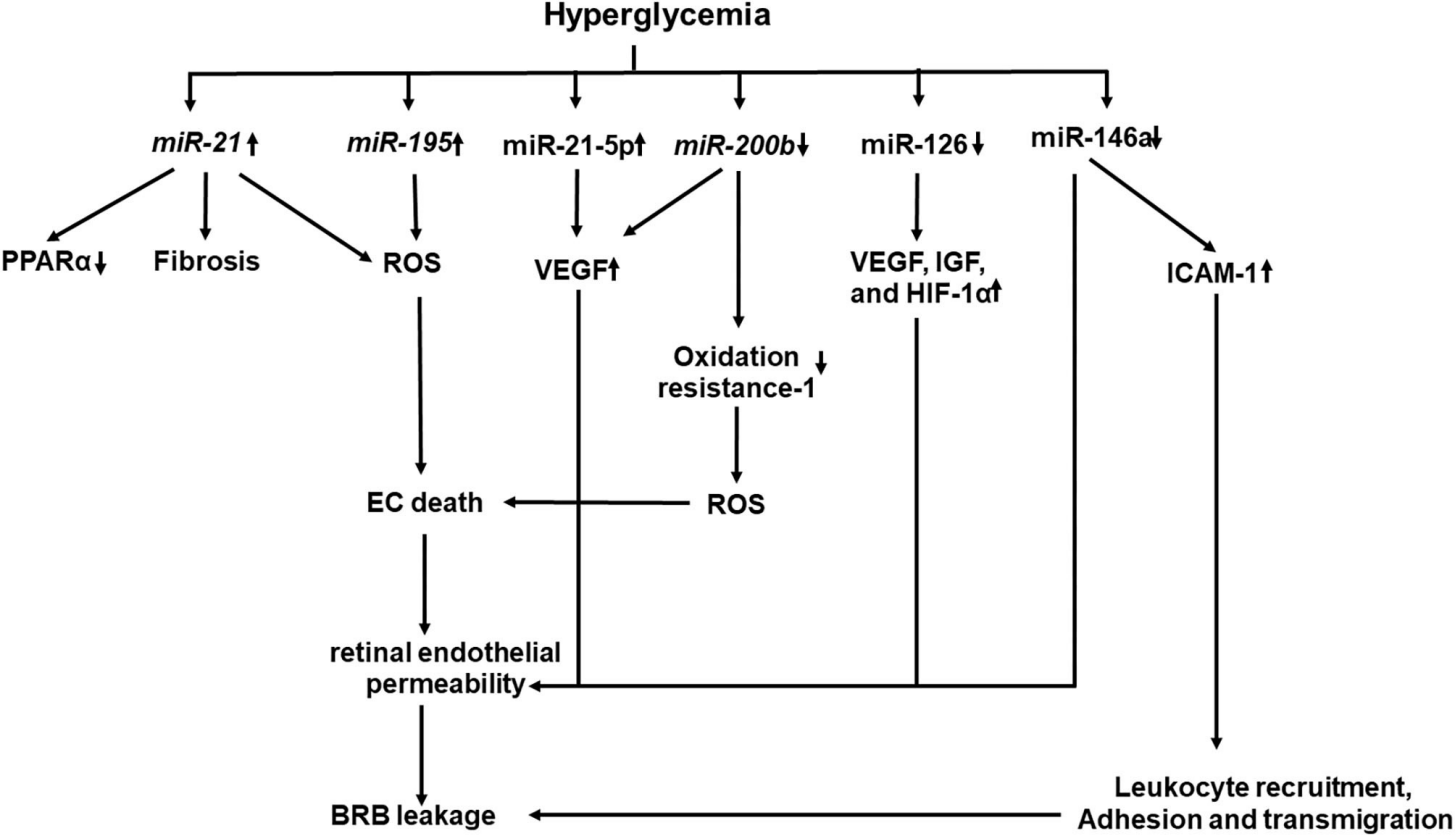
A schematic model of interaction networks mediated by miRNAs that contributes to blood retinal (BRB) leakage in diabetic retinopathy.

Recent studies have shown that epigenetics also plays a key role in the development and progression of DR (184–186). Hyperglycemia affects the enzymatic machinery responsible for epigenetic modifications (187). The enzymes responsible for epigenetic modifications and non-coding RNA function may be aberrantly expressed (Figure 4). They have been shown to either promote or inhibit the development and progression of DR (187). miRNAs and long non-coding RNA, which are well-known for their regulatory functions, are gaining more attention. Several studies identified panels of miRNAs whose expressions are changed in the retinal ECs of diabetic rats (184–186). NF-κB-responsive miRNA, such as *miR-21, miR-146, miR-155*, and *miR-132*, and VEGF-responsive miRNAs, such as *miR-17-5p, miR-18a, miR-20a, miR-21, miR-31*, and *miR-155*, have been identified in the retinal ECs (184). Wu et al. identified 11 increased miRNAs and 6 decreased miRNAs in the retinas of diabetic rats (185), while Xiong et al. identified 17 dysregulated miRNAs in the retinas of diabetic rats (186). Li et al. identified five differentially expressed miRNAs in serum between DR and non-DR patients (188). These miRNAs were found to regulate 55 target genes which were involved in controlling the vascular growth and morphogenesis.

### Upregulated miRNAS in DR

Increased miRNAs, such as miR-21 and miR-195, have been demonstrated to be associated with fibrosis and oxidative stress in DR (189, 190). Increased miR-21 level in the vitreous has been shown to be associated with retinal fibrosis in PDR (189). High glucose and TGF-β induce miR-21 expression in retinal pigment epithelial cells. Furthermore, gain and loss of function studies have shown that miR-21 promotes proliferation and migration of the human retinal pigment epithelium (189). *miR-21* affects PPARα expression through inhibition of PPARα mRNA translation (191). Intravitreal injection of the *miR-21* inhibitor attenuates PPARα downregulation and ameliorates retinal inflammation in db/db mice (191). Knockout of *miR-21* prevents the reduction of PPARα, which is associated with alleviated inflammation and microvascular damage in the retina of *db/db* mice. *miR-221* enhances retinal EC viability and angiogenesis through activation of PI3K/Akt/VEGF and inhibits the expression of PTEN (192). miR-21 downregulates the expression of Krev interaction trapped protein 1 (KRIT1), Nrf2, and SOD2, all of which are involved in ROS homeostasis, while the *miR-21* inhibitor improves KRIT1 and SOD2 expressions, reduces ROS production, and ameliorates mitochondrial membrane potential in HUVECs treated with high glucose (193). More recently, plasma *miR-21* has been proposed to be an early marker for diagnosis and identification of diabetic nephropathy in type 1 diabetes mellitus (T1DM), as it starts to rise before microalbuminuria in patients with T1DM and has a greater sensitivity (94.1%) and specificity (100%) to identify DN than the urinary albumin/creatinine ratio at level 45 mg/gm with sensitivity of 88.2% and specificity of 89% (194).

High glucose stimulates *miR-21-5p* expression, in parallel with increased VEGF and VEGFR2 expressions and proliferation of human retinal microvascular ECs (195). Inhibition of *miR-21-5p* reduces proliferation, migration, and tube formation of human retinal microvascular ECs (HRMECs) through PI3K/AKT and ERK pathways (195).

Upregulated miR-195 and downregulated SIRT1 have been observed in human retinal ECs exposed to high glucose and in the retinas of diabetic rats (190). Inhibition of miR-195 recovers SIRT1 expression and decreases retinal damage in DR (190). In addition, oxidative stress induces overexpression of miR-195 which downregulates mitofusin two expression in human retinal ECs and diabetic retinas, leading to increased permeability of retinal BRB (196).

### Downregulated miRNAs in DR

Decreased miRNAs, such as miR-126, *miR-146a*, and *miR-200b*, have been shown to increase the angiogenic factor product, promote the NF-κB pathway, and enhance VEGF-A expression and oxidative stress in DR, respectively. miR-126 is involved in the production of angiogenic factors to mediate retinal neovascularization (197, 198). A significant reduction of miR-126 in the serum is detected in patients with diabetes and macrovascular complications (199) or PDR (200). Downregulated *miR-126* is observed in the retinas of oxygen-induced retinopathy, while restoring its level reduces high expression levels of VEGF, IGF, and HIF-1α, which limits retinal neovascularization through p38MAPK and ERK pathways (197). *miR-126* is downregulated in hypoxia-treated rhesus retinal ECs and in retinas of diabetic rats, while restoring *miR-126* expression inhibits the hypoxia-induced neovascularization by inhibiting cell-cycle progression and the expression of VEGF and matrix metallopeptidase nine. Interestingly, hyperglycemic/hypoxia-treated mesenchymal stem cell-derived extracellular vesicles downregulate miR-126 in pericytes, which express more VEGF and HIF-1α (201).

*miR-146a* has a regulatory role in the NF-κB-mediated inflammatory pathway. It binds to the 3′-UTR of I IL-1 receptor-associated kinase 1 to reduce the expression of NF-κB-responsive ICAM-1 in both human retinal ECs and retinas of diabetic rats (202). Intravitreal delivery of *miR-146a* inhibits the hyperglycemia-induced upregulation of ICAM1 and reduces microvascular leakage and retinal functional defects. Increased miR-146a protects human retinal ECs from high glucose-induced apoptosis through suppressing the STAT3/VEGF pathway (203). Decreased miR-146a expression has been shown to be associated with the overexpression of fibronectin in high glucose-treated ECs and retinas of diabetic rats (204). Decreased *miR-146b-3p* has been shown to be associated with increased adenosine deaminase-2 (ADA-2) activity in the vitreous of patients with diabetes, while elevated expression of *miR-146b-3p* suppresses the ADA2 activity and TNF-α release in amadori-glycated albumin (AGA)-treated human macrophages (205) and decreases human retinal EC permeability and leukocyte adhesion by upregulating ICAM-1 (205).

Decreased *miR-200b* and increased VEGF-A gene expression were observed in the sera of patients with DR (206). Decreased *miR-200b* is observed in high glucose-treated human retinal ECs and is accompanied with increased expressions of VEGF and transforming growth factor β (206). Increased *miR-200b* expression inhibits the oxidation resistance one expression, which enhances resistance to apoptosis and oxidative stress (207).

A number of miRNAs have been investigated and are considered as a therapeutic target of DR. However, as a single miRNA can regulate several target genes that modulate different signaling pathways, miRNA-based therapy should be more refined and controlled for its targeting genes. The systematic understanding miRNA action mechanism may help for the early diagnosis and improved therapeutics for DR.

## Other Factors Contributing to or Associated With DR

In addition to the above discussed factors, recently studies identified new factors which may contribute to DR. Hyperglycemia induced circulating mitochondrial DNA change in parallel with increased circulating interleukin-4 and TNF-α in patients with DR, suggesting that mitochondrial DNA change in early diabetes may be an indicator of inflammation and progression of DR (208). Loukovaara et al. have found that the nucleotide-binding domain and leucine-rich repeat receptor containing pyrin domain 3 (NLRP3) inflammasome activation is associated with the vitreous pathogenesis of PDR (209).

Monosodium urate (MSU) has been found in human retinas and vitreous (210). Its level is correlated with inflammatory biomarkers and increased expression of xanthine oxidase (210). The MSU level is also detected in the serum and vitreous of diabetic rats and associated with NLRP3 inflammasome, suggesting a role of MSU in diabetes-induced retinal inflammation.

Shalaby et al. showed that a disintegrin and metalloprotease domain-17 (ADAM-17) might be involved in the development of DR (211). ADAM-17 activity is upregulated in human diabetic retinas and diabetic mouse. Loss of ADAM17 in ECs significantly reduces oxidative stress and decreases leukocyte adhesion *in vivo* and *in vitro*. Reduction in oxidative stress in retinal ECs is mediated by downregulation of NADPH oxidase 4 expression, while reduced leukostasis is achieved through downregulation of ICAM-1.

Asymmetric dimethylarginine level has elevated in both aqueous from diabetic rats and culture medium in rhesus retinal ECs pretreated with hypoxia (212). ADMA promotes proliferation, migration, adhesion, and tube formation of rhesus retinal ECs through the ephrin-B2 pathway.

## Challenges

Although we know that diabetes causes DR, not all patients after many years with diabetes develop DR, even in patients with poor glycemia. In contrast, some patients under good glycemic control still develop vision-threatening DR complications, indicating that we are still unclear what is the master regulator that initiates and controls the progress of diabetic retinopathy. VEGF has been proposed; however, a substantial proportion (40–50%) of patients with DME do not respond satisfactorily to anti-VEGF treatment (2).

Almost all cells in the retina can serve as effectors or donors of pro-inflammatory cytokines, VEGF, and ROS, through which to affect each other. Thus, it is difficult to dissect which cell type plays roles at which stage of DR. Knowing this is important and, as it may decide the outcome of treatment as various responses from different retinal cells, may abolish therapeutic effect.

The complex interactions among multiple contributors indicate that DR is a much more complicated disease situation. Treatment targeting at a single factor will be insufficient to reverse the progression of DR.

## Future Direction

Diabetes is a metabolic disease which is associated with lifestyle, environment, and genetics. Genetic factors may determine the discrepancy of DR morbidity and severity seen in patients with diabetes. A genome-wide association study associated, including RNA-seq, with metabolomics may be needed to identify signature genetic information associated with phenotypes seen in DR and metabolites. These techniques will not only enrich our understanding of the molecular mechanism in the initiation and progression of DR but also provide new molecular targets.

## Author Contributions

FG: conception and first draft and revision. ZY, SF, and HW: first draft of manuscript. YZ: revision. All authors: contributed to the article and approved the submitted version.

## Conflict of Interest

The authors declare that the research was conducted in the absence of any commercial or financial relationships that could be construed as a potential conflict of interest.

## References

[1] Gaborit B Julla JB Besbes S Proust M Vincentelli C Alos B. Glucagon-like peptide 1 receptor agonists, diabetic retinopathy and angiogenesis: the angiosafe type 2 diabetes study. J Clin Endocrinol Metab. (2020) 105:dgz069. 10.1210/clinem/dgz069 31589290

[2] Duh EJ Sun JK Stitt AW. Diabetic retinopathy: current understanding, mechanisms, and treatment strategies. JCI Insight. (2017) 2:e93751. 10.1172/jci.insight.93751 28724805 PMC5518557

[3] Lee R Wong TY Sabanayagam C. Epidemiology of diabetic retinopathy, diabetic macular edema and related vision loss. Eye Vis. (2015) 2:17. 10.1186/s40662-015-0026-2 26605370 PMC4657234

[4] Simo R Stitt AW Gardner TW. Neurodegeneration in diabetic retinopathy: does it really matter? Diabetologia. (2018) 61:1902–12. 10.1007/s00125-018-4692-1 30030554 PMC6096638

[5] Bandello F Lattanzio R Zucchiatti I Del Turco C. Pathophysiology and treatment of diabetic retinopathy. Acta Diabetol. (2013) 50:1–20. 10.1007/s00592-012-0449-3 23277338

[6] Williams R Airey M Baxter H Forrester J Kennedy-Martin T Girach A. Epidemiology of diabetic retinopathy and macular oedema: a systematic review. Eye. (2004) 18:963–83. 10.1038/sj.eye.6701476 15232600

[7] Yuuki T Kanda T Kimura Y Kotajima N Tamura J Kobayashi I Kishi S. Inflammatory cytokines in vitreous fluid and serum of patients with diabetic vitreoretinopathy. J Diabetes Complications. (2001) 15:257–9. 10.1016/S1056-8727(01)00155-6 11522500

[8] Miyamoto K Hiroshiba N Tsujikawa A Ogura Y. *In vivo* demonstration of increased leukocyte entrapment in retinal microcirculation of diabetic rats. Invest Ophthalmol Vis Sci. (1998) 39:2190–4. 9761301

[9] Rubsam A Parikh S Fort PE. Role of inflammation in diabetic retinopathy. Int J Mol Sci. (2018) 19:942. 10.3390/ijms19040942 29565290 PMC5979417

[10] Roy S Kern TS Song B Stuebe C. Mechanistic insights into pathological changes in the diabetic retina: implications for targeting diabetic retinopathy. Am J Pathol. (2017) 187:9–19. 10.1016/j.ajpath.2016.08.022 27846381 PMC5225303

[11] Antonetti DA Klein R Gardner TW. Diabetic retinopathy. N Engl J Med. (2012) 366:1227–39. 10.1056/NEJMra1005073 22455417

[12] Masuda T Shimazawa M Hara H. Retinal diseases associated with oxidative stress and the effects of a free radical scavenger (Edaravone). Oxid Med Cell Longev. (2017) 2017:9208489. 10.1155/2017/9208489 28194256 PMC5286467

[13] Wang J Xu X Elliott MH Zhu M Le YZ. Muller cell-derived VEGF is essential for diabetes-induced retinal inflammation and vascular leakage. Diabetes. (2010) 59:2297–305. 10.2337/db09-1420 20530741 PMC2927953

[14] Behl T Kaur I Kotwani A. Implication of oxidative stress in progression of diabetic retinopathy. Surv Ophthalmol. (2016) 61:187–96. 10.1016/j.survophthal.2015.06.001 26074354

[15] Tao D Ni N Zhang T Li C Sun Q Wang L Mei Y. Accumulation of advanced glycation end products potentiate human retinal capillary endothelial cells mediated diabetic retinopathy. Mol Med Rep. (2019) 20:3719–27. 10.3892/mmr.2019.10590 31432194 PMC6755164

[16] Xu J Chen LJ Yu J Wang HJ Zhang F Liu Q Wu J. Involvement of advanced glycation end products in the pathogenesis of diabetic retinopathy. Cell Physiol Biochem. (2018) 48:705–17. 10.1159/000491897 30025404

[17] Uribarri J Stirban A Sander D Cai W Negrean M Buenting CE. Single oral challenge by advanced glycation end products acutely impairs endothelial function in diabetic and nondiabetic subjects. Diabetes Care. (2007) 30:2579–82. 10.2337/dc07-0320 17496238

[18] Giardino I Edelstein D Brownlee M. Nonenzymatic glycosylation *in vitro* and in bovine endothelial cells alters basic fibroblast growth factor activity. A model for intracellular glycosylation in diabetes. J Clin Invest. (1994) 94:110–7. 10.1172/JCI117296 8040253 PMC296288

[19] Hsieh CL Yang MH Chyau CC Chiu CH Wang HE Lin YC. Kinetic analysis on the sensitivity of glucose- or glyoxal-induced LDL glycation to the inhibitory effect of *Psidium guajava* extract in a physiomimic system. Biosystems. (2007) 88:92–100. 10.1016/j.biosystems.2006.04.004 16806668

[20] Al-Mesallamy HO Hammad LN El-Mamoun TA Khalil BM. Role of advanced glycation end product receptors in the pathogenesis of diabetic retinopathy. J Diabetes Complications. (2011) 25:168–74. 10.1016/j.jdiacomp.2010.06.005 20685137

[21] Tanaka N Yonekura H Yamagishi S Fujimori H Yamamoto Y Yamamoto H. The receptor for advanced glycation end products is induced by the glycation products themselves and tumor necrosis factor-alpha through nuclear factor-kappa B, and by 17beta-estradiol through Sp-1 in human vascular endothelial cells. J Biol Chem. (2000) 275:25781–90. 10.1074/jbc.M001235200 10829018

[22] Yan SD Schmidt AM Anderson GM Zhang J Brett J Zou YS. Enhanced cellular oxidant stress by the interaction of advanced glycation end products with their receptors/binding proteins. J Biol Chem. (1994) 269:9889–97. 8144582

[23] Kandarakis SA Piperi C Topouzis F Papavassiliou AG. Emerging role of advanced glycation-end products (AGEs) in the pathobiology of eye diseases. Prog Retin Eye Res. (2014) 42:85–102. 10.1016/j.preteyeres.2014.05.002 24905859

[24] Yamagishi S Matsui T. Advanced glycation end products (AGEs), oxidative stress and diabetic retinopathy. Curr Pharm Biotechnol. (2011) 12:362–8. 10.2174/138920111794480534 20939798

[25] Bierhaus A Chevion S Chevion M Hofmann M Quehenberger P Illmer T. Advanced glycation end product-induced activation of NF-kappaB is suppressed by alpha-lipoic acid in cultured endothelial cells. Diabetes. (1997) 46:1481–90. 10.2337/diabetes.46.9.1481 9287050

[26] Schiekofer S Andrassy M Chen J Rudofsky G Schneider J Wendt T. Acute hyperglycemia causes intracellular formation of CML and activation of ras, p42/44 MAPK, and nuclear factor kappaB in PBMCs. Diabetes. (2003) 52:621–33. 10.2337/diabetes.52.3.621 12606501

[27] Drummond GR Selemidis S Griendling KK Sobey CG. Combating oxidative stress in vascular disease: NADPH oxidases as therapeutic targets. Nat Rev Drug Discov. (2011) 10:453–71. 10.1038/nrd3403 21629295 PMC3361719

[28] Schmidt AM Hori O Brett J Yan SD Wautier JL Stern D. Cellular receptors for advanced glycation end products. Implications for induction of oxidant stress and cellular dysfunction in the pathogenesis of vascular lesions. Arterioscler Thromb. (1994) 14:1521–8. 10.1161/01.ATV.14.10.1521 7918300

[29] Basta G Lazzerini G Del Turco S Ratto GM Schmidt AM De Caterina R. At least 2 distinct pathways generating reactive oxygen species mediate vascular cell adhesion molecule-1 induction by advanced glycation end products. Arterioscler Thromb Vasc Biol. (2005) 25:1401–7. 10.1161/01.ATV.0000167522.48370.5e 15845907

[30] Coughlan MT Thorburn DR Penfold SA Laskowski A Harcourt BE Sourris KC. RAGE-induced cytosolic ROS promote mitochondrial superoxide generation in diabetes. J Am Soc Nephrol. (2009) 20:742–52. 10.1681/ASN.2008050514 19158353 PMC2663823

[31] Morita M Yano S Yamaguchi T Sugimoto T. Advanced glycation end products-induced reactive oxygen species generation is partly through NF-kappa B activation in human aortic endothelial cells. J Diabetes Complications. (2013) 27:11–5. 10.1016/j.jdiacomp.2012.07.006 22944044

[32] Yamamoto Y Yamamoto H. Receptor for advanced glycation end-products-mediated inflammation and diabetic vascular complications. J Diabetes Investig. (2011) 2:155–7. 10.1111/j.2040-1124.2011.00125.x 24843476 PMC4014911

[33] Mamputu JC Renier G. Advanced glycation end products increase, through a protein kinase C-dependent pathway, vascular endothelial growth factor expression in retinal endothelial cells. Inhibitory effect of gliclazide. J Diabetes Complications. (2002) 16:284–93. 10.1016/S1056-8727(01)00229-X 12126787

[34] Rhee SY Kim YS. The role of advanced glycation end products in diabetic vascular complications. Diabetes Metab J. (2018) 42:188–95. 10.4093/dmj.2017.0105 29885110 PMC6015964

[35] Ai J Liu Y Sun JH. Advanced glycation end-products stimulate basic fibroblast growth factor expression in cultured muller cells. Mol Med Rep. (2013) 7:16–20. 10.3892/mmr.2012.1152 23129015 PMC3572729

[36] Frommhold D Kamphues A Hepper I Pruenster M Lukic IK Socher I. RAGE and ICAM-1 cooperate in mediating leukocyte recruitment during acute inflammation *in vivo*. Blood. (2010) 116:841–9. 10.1182/blood-2009-09-244293 20407037

[37] Mamputu JC Renier G. Advanced glycation end-products increase monocyte adhesion to retinal endothelial cells through vascular endothelial growth factor-induced ICAM-1 expression: inhibitory effect of antioxidants. J Leukoc Biol. (2004) 75:1062–9. 10.1189/jlb.0603265 15020646

[38] Joussen AM Poulaki V Mitsiades N Cai WY Suzuma I Pak J. Suppression of Fas-FasL-induced endothelial cell apoptosis prevents diabetic blood-retinal barrier breakdown in a model of streptozotocin-induced diabetes. FASEB J. (2003) 17:76–8. 10.1096/fj.02-0157fje 12475915

[39] van der Wijk AE Hughes JM Klaassen I Van Noorden CJF Schlingemann RO. Is leukostasis a crucial step or epiphenomenon in the pathogenesis of diabetic retinopathy? J Leukoc Biol. (2017) 102:993–1001. 10.1189/jlb.3RU0417-139 28724696

[40] Wu MH Ying NW Hong TM Chiang WF Lin YT Chen YL. Galectin-1 induces vascular permeability through the neuropilin-1/vascular endothelial growth factor receptor-1 complex. Angiogenesis. (2014) 17:839–49. 10.1007/s10456-014-9431-8 24719187

[41] Kanda A Dong Y Noda K Saito W Ishida S. Advanced glycation endproducts link inflammatory cues to upregulation of galectin-1 in diabetic retinopathy. Sci Rep. (2017) 7:16168. 10.1038/s41598-017-16499-8 29170525 PMC5700925

[42] De Silva TM Li Y Kinzenbaw DA Sigmund CD Faraci FM. Endothelial PPARgamma (peroxisome proliferator-activated receptor-gamma) is essential for preventing endothelial dysfunction with aging. Hypertension. (2018) 72:227–234. 10.1161/HYPERTENSIONAHA.117.10799 29735632 PMC6002945

[43] Chhabra M Sharma S. Potential role of peroxisome proliferator activated receptor gamma analogues in regulation of endothelial progenitor cells in diabetes mellitus: an overview. Diabetes Metab Syndr. (2019) 13:1123–9. 10.1016/j.dsx.2019.01.036 31336454

[44] Korbecki J Bobinski R Dutka M. Self-regulation of the inflammatory response by peroxisome proliferator-activated receptors. Inflamm Res. (2019) 68:443–58. 10.1007/s00011-019-01231-1 30927048 PMC6517359

[45] Wang F Gao L Gong B Hu J Li M Guan Q Zhao J. Tissue-specific expression of PPAR mRNAs in diabetic rats and divergent effects of cilostazol. Can J Physiol Pharmacol. (2008) 86:465–71. 10.1139/Y08-043 18641696

[46] Tawfik A Sanders T Kahook K Akeel S Elmarakby A Al-Shabrawey M. Suppression of retinal peroxisome proliferator-activated receptor gamma in experimental diabetes and oxygen-induced retinopathy: role of NADPH oxidase. Invest Ophthalmol Vis Sci. (2009) 50:878–84. 10.1167/iovs.08-2005 18806296

[47] Toda N Nakanishi-Toda M. Nitric oxide: ocular blood flow, glaucoma, and diabetic retinopathy. Prog Retin Eye Res. (2007) 26:205–38. 10.1016/j.preteyeres.2007.01.004 17337232

[48] Polikandriotis JA Mazzella LJ Rupnow HL Hart CM. Peroxisome proliferator-activated receptor gamma ligands stimulate endothelial nitric oxide production through distinct peroxisome proliferator-activated receptor gamma-dependent mechanisms. Arterioscler Thromb Vasc Biol. (2005) 25:1810–6. 10.1161/01.ATV.0000177805.65864.d4 16020752

[49] Shin E Yeo E Lim J Chang YH Park H Shim E. Nitrooleate mediates nitric oxide synthase activation in endothelial cells. Lipids. (2014) 49:457–66. 10.1007/s11745-014-3893-8 24664541

[50] Rudnicki M Tripodi GL Ferrer R Bosca L Pitta MG Pitta IR Abdalla DS. New thiazolidinediones affect endothelial cell activation and angiogenesis. Eur J Pharmacol. (2016) 782:98–106. 10.1016/j.ejphar.2016.04.038 27108791

[51] Hwang J Kleinhenz DJ Lassegue B Griendling KK Dikalov S Hart CM. Peroxisome proliferator-activated receptor-gamma ligands regulate endothelial membrane superoxide production. Am J Physiol Cell Physiol. (2005) 288:C899–905. 10.1152/ajpcell.00474.2004 15590897

[52] Werner CM Schirmer SH Gensch C Pavlickova V Poss J Wright MB. The dual PPARalpha/gamma agonist aleglitazar increases the number and function of endothelial progenitor cells: implications for vascular function and atherogenesis. Br J Pharmacol. (2014) 171:2685–703. 10.1111/bph.12608 24467636 PMC4009009

[53] Liang C Ren Y Tan H He Z Jiang Q Wu J. Rosiglitazone via upregulation of Akt/eNOS pathways attenuates dysfunction of endothelial progenitor cells, induced by advanced glycation end products. Br J Pharmacol. (2009) 158:1865–73. 10.1111/j.1476-5381.2009.00450.x 19917066 PMC2807648

[54] Blanquicett C Kang BY Ritzenthaler JD Jones DP Hart CM. Oxidative stress modulates PPAR gamma in vascular endothelial cells. Free Radic Biol Med. (2010) 48:1618–25. 10.1016/j.freeradbiomed.2010.03.007 20302927 PMC2868091

[55] Kim T Yang Q. Peroxisome-proliferator-activated receptors regulate redox signaling in the cardiovascular system. World J Cardiol. (2013) 5:164–74. 10.4330/wjc.v5.i6.164 23802046 PMC3691497

[56] Kronke G Kadl A Ikonomu E Bluml S Furnkranz A Sarembock IJ. Expression of heme oxygenase-1 in human vascular cells is regulated by peroxisome proliferator-activated receptors. Arterioscler Thromb Vasc Biol. (2007) 27:1276–82. 10.1161/ATVBAHA.107.142638 17413033

[57] Polvani S Tarocchi M Galli A. PPARgamma and oxidative stress: con (beta) catenating NRF2 and FOXO. PPAR Res. (2012) 2012:641087. 10.1155/2012/641087 22481913 PMC3317010

[58] Sena CM Matafome P Louro T Nunes E Fernandes R Seica RM. Metformin restores endothelial function in aorta of diabetic rats. Br J Pharmacol. (2011) 163:424–37. 10.1111/j.1476-5381.2011.01230.x 21250975 PMC3087142

[59] Chung SS Kim M Youn BS Lee NS Park JW Lee IK. Glutathione peroxidase 3 mediates the antioxidant effect of peroxisome proliferator-activated receptor gamma in human skeletal muscle cells. Mol Cell Biol. (2009) 29:20–30. 10.1128/MCB.00544-08 18936159 PMC2612482

[60] Inoue I Goto S Matsunaga T Nakajima T Awata T Hokari S. The ligands/activators for peroxisome proliferator-activated receptor alpha (PPARalpha) and PPARgamma increase Cu2+,Zn2+-superoxide dismutase and decrease p22phox message expressions in primary endothelial cells. Metabolism. (2001) 50:3–11. 10.1053/meta.2001.19415 11172467

[61] Sorrentino SA Bahlmann FH Besler C Muller M Schulz S Kirchhoff N. Oxidant stress impairs *in vivo* reendothelialization capacity of endothelial progenitor cells from patients with type 2 diabetes mellitus: restoration by the peroxisome proliferator-activated receptor-gamma agonist rosiglitazone. Circulation. (2007) 116:163–73. 10.1161/CIRCULATIONAHA.106.684381 17592079

[62] Liu L Wang Z Jia J Shi Y Lian T Han X. Linc01230, transcriptionally regulated by PPARgamma, is identified as a novel modifier in endothelial function. Biochem Biophys Res Commun. (2018) 507:369–76. 10.1016/j.bbrc.2018.11.045 30454889

[63] Lee C. Collaborative power of Nrf2 and PPARgamma activators against metabolic and drug-induced oxidative injury. Oxid Med Cell Longev. (2017) 2017:1378175. 10.1155/2017/1378175 28928902 PMC5591982

[64] Kim J Keum YS. NRF2, a key regulator of antioxidants with two faces towards cancer. Oxid Med Cell Longev. (2016) 2016:2746457. 10.1155/2016/2746457 27340506 PMC4909917

[65] Deliyanti D Alrashdi SF Tan SM Meyer C Ward KW de Haan JB Wilkinson-Berka JL. Nrf2 activation is a potential therapeutic approach to attenuate diabetic retinopathy. Invest Ophthalmol Vis Sci. (2018) 59:815–25. 10.1167/iovs.17-22920 29411009

[66] Jeong YH Park JS Kim DH Kim HS. Lonchocarpine Increases Nrf2/ARE-Mediated antioxidant enzyme expression by modulating AMPK and MAPK signaling in brain astrocytes. Biomol Ther (Seoul). (2016) 24:581–8. 10.4062/biomolther.2016.141 27737527 PMC5098536

[67] Huang J Tabbi-Anneni I Gunda V Wang L. Transcription factor Nrf2 regulates SHP and lipogenic gene expression in hepatic lipid metabolism. Am J Physiol Gastrointest Liver Physiol. (2010) 299:G1211–21. 10.1152/ajpgi.00322.2010 20930048 PMC3006243

[68] Park EY Cho IJ Kim SG. Transactivation of the PPAR-responsive enhancer module in chemopreventive glutathione S-transferase gene by the peroxisome proliferator-activated receptor-gamma and retinoid X receptor heterodimer. Cancer Res. (2004) 64:3701–13. 10.1158/0008-5472.CAN-03-3924 15150131

[69] Semeraro F Morescalchi F Cancarini A Russo A Rezzola S Costagliola C. Diabetic retinopathy, a vascular and inflammatory disease: therapeutic implications. Diabetes Metab. (2019) 45:517–27. 10.1016/j.diabet.2019.04.002 31005756

[70] Mesquida M Drawnel F Fauser S. The role of inflammation in diabetic eye disease. Semin Immunopathol. (2019) 41:427–45. 10.1007/s00281-019-00750-7 31175392

[71] Semeraro F Cancarini A dell'Omo R Rezzola S Romano MR Costagliola C. Diabetic retinopathy: vascular and inflammatory disease. J Diabetes Res. (2015) 2015:582060. 10.1155/2015/582060 26137497 PMC4475523

[72] dell'Omo R Semeraro F Bamonte G Cifariello F Romano MR Costagliola C. Vitreous mediators in retinal hypoxic diseases. Mediators Inflamm. (2013) 2013:935301. 10.1155/2013/935301 23365490 PMC3556845

[73] Abcouwer SF. Angiogenic factors and cytokines in diabetic retinopathy. J Clin Cell Immunol. (2013) (Suppl. 1):1–12. 10.4172/2155-9899 24319628 PMC3852182

[74] Patel JI Saleh GM Hykin PG Gregor ZJ Cree IA. Concentration of haemodynamic and inflammatory related cytokines in diabetic retinopathy. Eye. (2008) 22:223–8. 10.1038/sj.eye.6702584 17001327

[75] Wu H Hwang DK Song X Tao Y. Association between aqueous cytokines and diabetic retinopathy stage. J Ophthalmol. (2017) 2017:9402198. 10.1155/2017/9402198 28680705 PMC5478856

[76] Kalliolias GD Ivashkiv LB. TNF biology, pathogenic mechanisms and emerging therapeutic strategies. Nat Rev Rheumatol. (2016) 12:49–62. 10.1038/nrrheum.2015.169 26656660 PMC4809675

[77] Yao Y Li R Du J Li X Zhao L Long L. Tumor necrosis factor-alpha and diabetic retinopathy: review and meta-analysis. Clin Chim Acta. (2018) 485:210–7. 10.1016/j.cca.2018.06.028 29959897

[78] Doganay S Evereklioglu C Er H Turkoz Y Sevinc A Mehmet N Savli H. Comparison of serum NO, TNF-alpha, IL-1beta, sIL-2R, IL-6 and IL-8 levels with grades of retinopathy in patients with diabetes mellitus. Eye. (2002) 16:163–70. 10.1038/sj/eye/6700095 11988817

[79] Costagliola C Romano V De Tollis M Aceto F dell'Omo R Romano MR. TNF-alpha levels in tears: a novel biomarker to assess the degree of diabetic retinopathy. Mediators Inflamm. (2013) 2013:629529. 10.1155/2013/629529 24259948 PMC3821908

[80] Aveleira CA Lin CM Abcouwer SF Ambrosio AF Antonetti DA. TNF-alpha signals through PKCzeta/NF-kappaB to alter the tight junction complex and increase retinal endothelial cell permeability. Diabetes. (2010) 59:2872–82. 10.2337/db09-1606 20693346 PMC2963546

[81] Huang H Gandhi JK Zhong X Wei Y Gong J Duh EJ Vinores SA. TNFalpha is required for late BRB breakdown in diabetic retinopathy, and its inhibition prevents leukostasis and protects vessels and neurons from apoptosis. Invest Ophthalmol Vis Sci. (2011) 52:1336–44. 10.1167/iovs.10-5768 21212173 PMC3101693

[82] Boss JD Singh PK Pandya HK Tosi J Kim C Tewari A. Assessment of neurotrophins and inflammatory mediators in vitreous of patients with diabetic retinopathy. Invest Ophthalmol Vis Sci. (2017) 58:5594–603. 10.1167/iovs.17-21973 29084332 PMC5667399

[83] Wooff Y Man SM Aggio-Bruce R Natoli R Fernando N. IL-1 family members mediate cell death, inflammation and angiogenesis in retinal degenerative diseases. Front Immunol. (2019) 10:1618. 10.3389/fimmu.2019.01618 31379825 PMC6646526

[84] Demircan N Safran BG Soylu M Ozcan AA Sizmaz S. Determination of vitreous interleukin-1 (IL-1) and tumour necrosis factor (TNF) levels in proliferative diabetic retinopathy. Eye. (2006) 20:1366–9. 10.1038/sj.eye.6702138 16284605

[85] Kowluru RA Odenbach S. Role of interleukin-1beta in the development of retinopathy in rats: effect of antioxidants. Invest Ophthalmol Vis Sci. (2004) 45:4161–6. 10.1167/iovs.04-0633 15505070

[86] Mendiola AS Cardona AE. The IL-1beta phenomena in neuroinflammatory diseases. J Neural Transm (Vienna). (2018) 125:781–95. 10.1007/s00702-017-1732-9 28534174 PMC5699978

[87] Zhou TE Rivera JC Bhosle VK Lahaie I Shao Z Tahiri H. Choroidal involution is associated with a progressive degeneration of the outer retinal function in a model of retinopathy of prematurity: early role for IL-1beta. Am J Pathol. (2016) 186:3100–16. 10.1016/j.ajpath.2016.08.004 27768863

[88] Liu X Ye F Xiong H Hu D Limb GA Xie T. IL-1beta upregulates IL-8 production in human muller cells through activation of the p38 MAPK and ERK1/2 signaling pathways. Inflammation. (2014) 37:1486–95. 10.1007/s10753-014-9874-5 24706000

[89] Liu X Ye F Xiong H Hu DN Limb GA Xie T. IL-1beta induces IL-6 production in retinal muller cells predominantly through the activation of p38 MAPK/NF-kappaB signaling pathway. Exp Cell Res. (2015) 331:223–31. 10.1016/j.yexcr.2014.08.040 25239226

[90] Liu Y Biarnes Costa M Gerhardinger C. IL-1beta is upregulated in the diabetic retina and retinal vessels: cell-specific effect of high glucose and IL-1beta autostimulation. PLoS ONE. (2012) 7:e36949. 10.1371/journal.pone.0036949 22615852 PMC3353989

[91] Koenig RT Dickman JR Kang CH Zhang T Chu YF Ji LL. Avenanthramide supplementation attenuates eccentric exercise-inflicted blood inflammatory markers in women. Eur J Appl Physiol. (2016) 116:67–76. 10.1007/s00421-015-3244-3 26289619

[92] Uciechowski P Dempke WCM. Interleukin-6: a masterplayer in the cytokine network. Oncology. (2020) 98:131–7. 10.1159/000505099 31958792

[93] Mocan MC Kadayifcilar S Eldem B. Elevated intravitreal interleukin-6 levels in patients with proliferative diabetic retinopathy. Can J Ophthalmol. (2006) 41:747–52. 10.3129/i06-070 17224958

[94] Yao Y Li R Du J Long L Li X Luo N. Interleukin-6 and diabetic retinopathy: a systematic review and meta-analysis. Curr Eye Res. (2019) 44:564–74. 10.1080/02713683.2019.1570274 30644770

[95] Rusnak S Vrzalova J Sobotova M Hecova L Ricarova R Topolcan O. The measurement of intraocular biomarkers in various stages of proliferative diabetic retinopathy using multiplex xMAP technology. J Ophthalmol. (2015) 2015:424783. 10.1155/2015/424783 26491551 PMC4600494

[96] Chalam KV Grover S Sambhav K Balaiya S Murthy RK. Aqueous interleukin-6 levels are superior to vascular endothelial growth factor in predicting therapeutic response to bevacizumab in age-related macular degeneration. J Ophthalmol. (2014) 2014:502174. 10.1155/2014/502174 25110587 PMC4121253

[97] Arjamaa O Pollonen M Kinnunen K Ryhanen T Kaarniranta K. Increased IL-6 levels are not related to NF-kappaB or HIF-1alpha transcription factors activity in the vitreous of proliferative diabetic retinopathy. J Diabetes Complications. (2011) 25:393–7. 10.1016/j.jdiacomp.2011.06.002 21813290

[98] Gao X Li Y Wang H Li C Ding J. Inhibition of HIF-1alpha decreases expression of pro-inflammatory IL-6 and TNF-alpha in diabetic retinopathy. Acta Ophthalmol. (2017) 95:e746–50. 10.1111/aos.13096 27288252

[99] Chen H Zhang X Liao N Wen F. Assessment of biomarkers using multiplex assays in aqueous humor of patients with diabetic retinopathy. BMC Ophthalmol. (2017) 17:176. 10.1186/s12886-017-0572-6 28969616 PMC5625688

[100] Petrovic MG Korosec P Kosnik M Hawlina M. Association of preoperative vitreous IL-8 and VEGF levels with visual acuity after vitrectomy in proliferative diabetic retinopathy. Acta Ophthalmol. (2010) 88:e311–6. 10.1111/j.1755-3768.2010.02030.x 21073666

[101] Wu F Phone A Lamy R Ma D Laotaweerungsawat S Chen Y. Correlation of aqueous, vitreous, and plasma cytokine levels in patients with proliferative diabetic retinopathy. Invest Ophthalmol Vis Sci. (2020) 61:26. 10.1167/iovs.61.2.26 32084272 PMC7326572

[102] Lee TH Avraham H Lee SH Avraham S. Vascular endothelial growth factor modulates neutrophil transendothelial migration via up-regulation of interleukin-8 in human brain microvascular endothelial cells. J Biol Chem. (2002) 277:10445–51. 10.1074/jbc.M107348200 11784713

[103] Feuerer M Eulenburg K Loddenkemper C Hamann A Huehn J. Self-limitation of Th1-mediated inflammation by IFN-gamma. J Immunol. (2006) 176:2857–63. 10.4049/jimmunol.176.5.2857 16493042

[104] Vujosevic S Micera A Bini S Berton M Esposito G Midena E. Proteome analysis of retinal glia cells-related inflammatory cytokines in the aqueous humour of diabetic patients. Acta Ophthalmol. (2016) 94:56–64. 10.1111/aos.12812 26268591

[105] Tsai T Kuehn S Tsiampalis N Vu MK Kakkassery V Stute G. Anti-inflammatory cytokine and angiogenic factors levels in vitreous samples of diabetic retinopathy patients. PLoS ONE. (2018) 13:e0194603. 10.1371/journal.pone.0194603 29584759 PMC5870958

[106] Johnsen-Soriano S Sancho-Tello M Arnal E Navea A Cervera E Bosch-Morell F. IL-2 and IFN-gamma in the retina of diabetic rats. Graefes Arch Clin Exp Ophthalmol. (2010) 248:985–90. 10.1007/s00417-009-1289-x 20213480

[107] Zinkernagel MS Chinnery HR Ong ML Petitjean C Voigt V McLenachan S. Interferon gamma-dependent migration of microglial cells in the retina after systemic cytomegalovirus infection. Am J Pathol. (2013) 182:875–85. 10.1016/j.ajpath.2012.11.031 23313136

[108] Geiger K Howes E Gallina M Huang XJ Travis GH Sarvetnick N. Transgenic mice expressing IFN-gamma in the retina develop inflammation of the eye and photoreceptor loss. Invest Ophthalmol Vis Sci. (1994) 35:2667–81. 8188461

[109] Ng CT Fong LY Low YY Ban J Hakim MN Ahmad Z. Nitric oxide participates in IFN-gamma-induced HUVECs hyperpermeability. Physiol Res. (2016) 65:1053–1058. 10.33549/physiolres.933237 27539106

[110] Bonney S Seitz S Ryan CA Jones KL Clarke P Tyler KL Siegenthaler JA. Gamma interferon alters junctional integrity via rho kinase, resulting in blood-brain barrier leakage in experimental viral encephalitis. mBio. (2019) 10:e01675–19. 10.1128/mBio.01675-19 31387911 PMC6686045

[111] Nahomi RB Palmer A Green KM Fort PE Nagaraj RH. Pro-inflammatory cytokines downregulate Hsp27 and cause apoptosis of human retinal capillary endothelial cells. Biochim Biophys Acta. (2014) 1842:164–74. 10.1016/j.bbadis.2013.11.011 24252613 PMC3905326

[112] Abcouwer SF. Muller cell-microglia cross talk drives neuroinflammation in diabetic retinopathy. Diabetes. (2017) 66:261–3. 10.2337/dbi16-0047 28108606 PMC5248992

[113] Dong N Chang L Wang B Chu L. Retinal neuronal MCP-1 induced by AGEs stimulates TNF-alpha expression in rat microglia via p38, ERK, and NF-kappaB pathways. Mol Vis. (2014) 20:616–28. 24826069 PMC4016805

[114] Gharaee-Kermani M Denholm EM Phan SH. Costimulation of fibroblast collagen and transforming growth factor beta1 gene expression by monocyte chemoattractant protein-1 via specific receptors. J Biol Chem. (1996) 271:17779–84. 10.1074/jbc.271.30.17779 8663511

[115] Taghavi Y Hassanshahi G Kounis NG Koniari I Khorramdelazad H. Monocyte chemoattractant protein-1 (MCP-1/CCL2) in diabetic retinopathy: latest evidence and clinical considerations. J Cell Commun Signal. (2019) 13:451–62. 10.1007/s12079-018-00500-8 30607767 PMC6946768

[116] Feng C Wang X Liu T Zhang M Xu G Ni Y. Expression of CCL2 and its receptor in activation and migration of microglia and monocytes induced by photoreceptor apoptosis. Mol Vis. (2017) 23:765–77. 29142497 PMC5669614

[117] Reddy S Amutha A Rajalakshmi R Bhaskaran R Monickaraj F Rangasamy S. Association of increased levels of MCP-1 and cathepsin-D in young onset type 2 diabetes patients (T2DM-Y) with severity of diabetic retinopathy. J Diabetes Complications. (2017) 31:804–9. 10.1016/j.jdiacomp.2017.02.017 28336215

[118] Rangasamy S McGuire PG Franco Nitta C Monickaraj F Oruganti SR Das A. Chemokine mediated monocyte trafficking into the retina: role of inflammation in alteration of the blood-retinal barrier in diabetic retinopathy. PLoS ONE. (2014) 9:e108508. 10.1371/journal.pone.0108508 25329075 PMC4203688

[119] Yoshida S Yoshida A Ishibashi T. Induction of IL-8, MCP-1, and bFGF by TNF-alpha in retinal glial cells: implications for retinal neovascularization during post-ischemic inflammation. Graefes Arch Clin Exp Ophthalmol. (2004) 242:409–13. 10.1007/s00417-004-0874-2 15029502

[120] Murugeswari P Shukla D Rajendran A Kim R Namperumalsamy P Muthukkaruppan V. Proinflammatory cytokines and angiogenic and anti-angiogenic factors in vitreous of patients with proliferative diabetic retinopathy and eales' disease. Retina. (2008) 28:817–24. 10.1097/IAE.0b013e31816576d5 18536597

[121] Krady JK Basu A Allen CM Xu Y LaNoue KF Gardner TW Levison SW. Minocycline reduces proinflammatory cytokine expression, microglial activation, and caspase-3 activation in a rodent model of diabetic retinopathy. Diabetes. (2005) 54:1559–65. 10.2337/diabetes.54.5.1559 15855346

[122] Nakazawa T Hisatomi T Nakazawa C Noda K Maruyama K She H. Monocyte chemoattractant protein 1 mediates retinal detachment-induced photoreceptor apoptosis. Proc Natl Acad Sci USA. (2007) 104:2425–30. 10.1073/pnas.0608167104 17284607 PMC1892947

[123] Hong KH Ryu J Han KH. Monocyte chemoattractant protein-1-induced angiogenesis is mediated by vascular endothelial growth factor-A. Blood. (2005) 105:1405–7. 10.1182/blood-2004-08-3178 15498848

[124] Suzuki Y Suzuki K Kudo T Metoki T Nakazawa M. Level of vascular endothelial growth factor in the vitreous fluid of proliferative diabetic retinopathy patients and prognosis after vitrectomy. Ophthalmologica. (2016) 236:133–8. 10.1159/000449261 27794575

[125] Abu El-Asrar AM Struyf S Mohammad G Gouwy M Rytinx P Siddiquei MM. Osteoprotegerin is a new regulator of inflammation and angiogenesis in proliferative diabetic retinopathy. Invest Ophthalmol Vis Sci. (2017) 58:3189–201. 10.1167/iovs.16-20993 28654984

[126] Chen B He T Xing Y Cao T. Effects of quercetin on the expression of MCP-1, MMP-9 and VEGF in rats with diabetic retinopathy. Exp Ther Med. (2017) 14:6022–6. 10.3892/etm.2017.5275 29285153 PMC5740807

[127] Yu Y Zhang J Zhu R Zhao R Chen J Jin J. The profile of angiogenic factors in vitreous humor of the patients with proliferative diabetic retinopathy. Curr Mol Med. (2017) 17:280–6. 10.2174/1566524017666171106111440 29110608

[128] Mitamura Y Harada C Harada T. Role of cytokines and trophic factors in the pathogenesis of diabetic retinopathy. Curr Diabetes Rev. (2005) 1:73–81. 10.2174/1573399052952596 18220584

[129] Freyberger H Brocker M Yakut H Hammer J Effert R Schifferdecker E. Increased levels of platelet-derived growth factor in vitreous fluid of patients with proliferative diabetic retinopathy. Exp Clin Endocrinol Diabetes. (2000) 108:106–9. 10.1055/s-2000-5803 10826517

[130] Shaw SK Ma S Kim MB Rao RM Hartman CU Froio RM. Coordinated redistribution of leukocyte LFA-1 and endothelial cell ICAM-1 accompany neutrophil transmigration. J Exp Med. (2004) 200:1571–80. 10.1084/jem.20040965 15611287 PMC2212000

[131] Dumas E Neagoe PE McDonald PP White M Sirois MG. New insights into the pro-inflammatory activities of Ang1 on neutrophils: induction of MIP-1beta synthesis and release. PLoS ONE. (2016) 11:e0163140. 10.1371/journal.pone.0163140 27632174 PMC5025150

[132] Poulaki V Joussen AM Mitsiades N Mitsiades CS Iliaki EF Adamis AP. Insulin-like growth factor-I plays a pathogenetic role in diabetic retinopathy. Am J Pathol. (2004) 165:457–69. 10.1016/S0002-9440(10)63311-1 15277220 PMC1618554

[133] Zhao LQ Cheng JW. A systematic review and meta-analysis of clinical outcomes of intravitreal Anti-VEGF agent treatment immediately after cataract surgery for patients with diabetic retinopathy. J Ophthalmol. (2019) 2019:2648267. 10.1155/2019/2648267 31143469 PMC6501156

[134] Shin ES Huang Q Gurel Z Sorenson CM Sheibani N. High glucose alters retinal astrocytes phenotype through increased production of inflammatory cytokines and oxidative stress. PLoS ONE. (2014) 9:e103148. 10.1371/journal.pone.0103148 25068294 PMC4113377

[135] Gerhardinger C Costa MB Coulombe MC Toth I Hoehn T Grosu P. Expression of acute-phase response proteins in retinal muller cells in diabetes. Invest Ophthalmol Vis Sci. (2005) 46:349–57. 10.1167/iovs.04-0860 15623795

[136] Zeng HY Green WR Tso MO. Microglial activation in human diabetic retinopathy. Arch Ophthalmol. (2008) 126:227–32. 10.1001/archophthalmol.2007.65 18268214

[137] Gariano RF Gardner TW. Retinal angiogenesis in development and disease. Nature. (2005) 438:960–6. 10.1038/nature04482 16355161

[138] Sone H Kawakami Y Okuda Y Kondo S Hanatani M Suzuki H Yamashita K. Vascular endothelial growth factor is induced by long-term high glucose concentration and up-regulated by acute glucose deprivation in cultured bovine retinal pigmented epithelial cells. Biochem Biophys Res Commun. (1996) 221:193–8. 10.1006/bbrc.1996.0568 8660335

[139] Gupta N Mansoor S Sharma A Sapkal A Sheth J Falatoonzadeh P. Diabetic retinopathy and VEGF. Open Ophthalmol J. (2013) 7:4–10. 10.2174/1874364101307010004 23459241 PMC3580758

[140] Wang X Wang G Wang Y. Intravitreous vascular endothelial growth factor and hypoxia-inducible factor 1a in patients with proliferative diabetic retinopathy. Am J Ophthalmol. (2009) 148:883–9. 10.1016/j.ajo.2009.07.007 19837381

[141] Matsuoka M Ogata N Minamino K Matsumura M. Expression of pigment epithelium-derived factor and vascular endothelial growth factor in fibrovascular membranes from patients with proliferative diabetic retinopathy. Jpn J Ophthalmol. (2006) 50:116–20. 10.1007/s10384-005-0294-9 16604386

[142] Wang J Chen S Jiang F You C Mao C Yu J. Vitreous and plasma VEGF levels as predictive factors in the progression of proliferative diabetic retinopathy after vitrectomy. PLoS ONE. (2014) 9:e110531. 10.1371/journal.pone.0110531 25329921 PMC4199758

[143] Baharivand N Zarghami N Panahi F Dokht Ghafari MY Mahdavi Fard A Mohajeri A. Relationship between vitreous and serum vascular endothelial growth factor levels, control of diabetes and microalbuminuria in proliferative diabetic retinopathy. Clin Ophthalmol. (2012) 6:185–91. 10.2147/OPTH.S27423 22331976 PMC3273407

[144] Adamis AP Miller JW Bernal MT D'Amico DJ Folkman J Yeo TK Yeo KT. Increased vascular endothelial growth factor levels in the vitreous of eyes with proliferative diabetic retinopathy. Am J Ophthalmol. (1994) 118:445–50. 10.1016/S0002-9394(14)75794-0 7943121

[145] Shinoda K Ishida S Kawashima S Wakabayashi T Matsuzaki T Takayama M. Comparison of the levels of hepatocyte growth factor and vascular endothelial growth factor in aqueous fluid and serum with grades of retinopathy in patients with diabetes mellitus. Br J Ophthalmol. (1999) 83:834–7. 10.1136/bjo.83.7.834 10381671 PMC1723111

[146] Mitamura Y Tashimo A Nakamura Y Tagawa H Ohtsuka K Mizue Y Nishihira J. Vitreous levels of placenta growth factor and vascular endothelial growth factor in patients with proliferative diabetic retinopathy. Diabetes Care. (2002) 25:2352. 10.2337/diacare.25.12.2352 12453985

[147] Abu El-Asrar AM Nawaz MI Kangave D Mairaj Siddiquei M Geboes K. Angiogenic and vasculogenic factors in the vitreous from patients with proliferative diabetic retinopathy. J Diabetes Res. (2013) 2013:539658. 10.1155/2013/539658 23671874 PMC3647558

[148] Ahuja S Saxena S Akduman L Meyer CH Kruzliak P Khanna VK. Serum vascular endothelial growth factor is a biomolecular biomarker of severity of diabetic retinopathy. Int J Retina Vitreous. (2019) 5:29. 10.1186/s40942-019-0179-6 31583119 PMC6771093

[149] Jain A Saxena S Khanna VK Shukla RK Meyer CH. Status of serum VEGF and ICAM-1 and its association with external limiting membrane and inner segment-outer segment junction disruption in type 2 diabetes mellitus. Mol Vis. (2013) 19:1760–8. 23922493 PMC3733909

[150] Zhang D Lv FL Wang GH. Effects of HIF-1alpha on diabetic retinopathy angiogenesis and VEGF expression. Eur Rev Med Pharmacol Sci. (2018) 22:5071–6. 10.26355/eurrev_201808_15699 30178824

[151] Tolentino MJ McLeod DS Taomoto M Otsuji T Adamis AP Lutty GA. Pathologic features of vascular endothelial growth factor-induced retinopathy in the nonhuman primate. Am J Ophthalmol. (2002) 133:373–85. 10.1016/S0002-9394(01)01381-2 11860975

[152] Joussen AM Poulaki V Qin W Kirchhof B Mitsiades N Wiegand SJ. Retinal vascular endothelial growth factor induces intercellular adhesion molecule-1 and endothelial nitric oxide synthase expression and initiates early diabetic retinal leukocyte adhesion *in vivo*. Am J Pathol. (2002) 160:501–9. 10.1016/S0002-9440(10)64869-9 11839570 PMC1850650

[153] Marumo T Schini-Kerth VB Busse R. Vascular endothelial growth factor activates nuclear factor-kappaB and induces monocyte chemoattractant protein-1 in bovine retinal endothelial cells. Diabetes. (1999) 48:1131–7. 10.2337/diabetes.48.5.1131 10331420

[154] Choudhuri S Chowdhury IH Das S Dutta D Saha A Sarkar R. Role of NF-kappaB activation and VEGF gene polymorphisms in VEGF up regulation in non-proliferative and proliferative diabetic retinopathy. Mol Cell Biochem. (2015) 405:265–79. 10.1007/s11010-015-2417-z 25956512

[155] Melder RJ Koenig GC Witwer BP Safabakhsh N Munn LL Jain RK. During angiogenesis, vascular endothelial growth factor and basic fibroblast growth factor regulate natural killer cell adhesion to tumor endothelium. Nat Med. (1996) 2:992–7. 10.1038/nm0996-992 8782456

[156] Zhang XL Wen L Chen YJ Zhu Y. Vascular endothelial growth factor up-regulates the expression of intracellular adhesion molecule-1 in retinal endothelial cells via reactive oxygen species, but not nitric oxide. Chin Med J. (2009) 122:338–43. 19236815

[157] Nakagawa T Tanabe K Croker BP Johnson RJ Grant MB Kosugi T Li Q. Endothelial dysfunction as a potential contributor in diabetic nephropathy. Nat Rev Nephrol. (2011) 7:36–44. 10.1038/nrneph.2010.152 21045790 PMC3653134

[158] Mitroulis I Alexaki VI Kourtzelis I Ziogas A Hajishengallis G Chavakis T. Leukocyte integrins: role in leukocyte recruitment and as therapeutic targets in inflammatory disease. Pharmacol Ther. (2015) 147:123–35. 10.1016/j.pharmthera.2014.11.008 25448040 PMC4324083

[159] Nourshargh S Hordijk PL Sixt M. Breaching multiple barriers: leukocyte motility through venular walls and the interstitium. Nat Rev Mol Cell Biol. (2010) 11:366–78. 10.1038/nrm2889 20414258

[160] Muller WA. How endothelial cells regulate transmigration of leukocytes in the inflammatory response. Am J Pathol. (2014) 184:886–96. 10.1016/j.ajpath.2013.12.033 24655376 PMC3969991

[161] Khalfaoui T Lizard G Ouertani-Meddeb A. Adhesion molecules (ICAM-1 and VCAM-1) and diabetic retinopathy in type 2 diabetes. J Mol Histol. (2008) 39:243–9. 10.1007/s10735-007-9159-5 18165914

[162] Nowak M Wielkoszynski T Marek B Kos-Kudla B Swietochowska E Sieminska L. Blood serum levels of vascular cell adhesion molecule (sVCAM-1), intercellular adhesion molecule (sICAM-1) and endothelial leucocyte adhesion molecule-1 (ELAM-1) in diabetic retinopathy. Clin Exp Med. (2008) 8:159–64. 10.1007/s10238-008-0173-z 18791689

[163] Yoshizawa M Nagai Y Ohsawa K Ohta M Yamashita H Hisada A. Elevated serum levels of soluble vascular cell adhesion molecule-1 in NIDDM patients with proliferative diabetic retinopathy. Diabetes Res Clin Pract. (1998) 42:65–70. 10.1016/S0168-8227(98)00091-6 9884035

[164] Okada M Matsuto T Miida T Inano K. Differences in the effects of cytokines on the expression of adhesion molecules in endothelial cells. Ann Med Interne. (1997) 148:125–9. 9238436

[165] Chen W Esselman WJ Jump DB Busik JV. Anti-inflammatory effect of docosahexaenoic acid on cytokine-induced adhesion molecule expression in human retinal vascular endothelial cells. Invest Ophthalmol Vis Sci. (2005) 46:4342–7. 10.1167/iovs.05-0601 16249517 PMC1378111

[166] Cao Y Feng B Chen S Chu Y Chakrabarti S. Mechanisms of endothelial to mesenchymal transition in the retina in diabetes. Invest Ophthalmol Vis Sci. (2014) 55:7321–31. 10.1167/iovs.14-15167 25335984

[167] Muller WA. Leukocyte-endothelial-cell interactions in leukocyte transmigration and the inflammatory response. Trends Immunol. (2003) 24:327–34. 10.1016/S1471-4906(03)00117-0 12810109

[168] Murata M Noda K Kawasaki A Yoshida S Dong Y Saito M. Soluble vascular adhesion protein-1 mediates spermine oxidation as semicarbazide-sensitive amine oxidase: possible role in proliferative diabetic retinopathy. Curr Eye Res. (2017) 42:1674–83. 10.1080/02713683.2017.1359847 28937866

[169] Subauste CS. CD40, a novel inducer of purinergic signaling: implications to the pathogenesis of experimental diabetic retinopathy. Vision. (2017) 1:30. 10.3390/vision1030020 31740645 PMC6835793

[170] van Kooten C Banchereau J. CD40-CD40 ligand. J Leukoc Biol. (2000) 67:2–17. 10.1002/jlb.67.1.2 10647992

[171] Shoelson SE Lee J Goldfine AB. Inflammation and insulin resistance. J Clin Invest. (2006) 116:1793–801. 10.1172/JCI29069 16823477 PMC1483173

[172] Stark K Eckart A Haidari S Tirniceriu A Lorenz M von Bruhl ML. Capillary and arteriolar pericytes attract innate leukocytes exiting through venules and 'instruct' them with pattern-recognition and motility programs. Nat Immunol. (2013) 14:41–51. 10.1038/ni.2477 23179077

[173] Lamine LB Turki A Al-Khateeb G Sellami N Amor HB Sarray S. Elevation in circulating soluble CD40 ligand concentrations in type 2 diabetic retinopathy and association with its severity. Exp Clin Endocrinol Diabetes. (2018) 128:319–24. 10.1055/a-0647-6860 30149416

[174] Portillo JA Greene JA Okenka G Miao Y Sheibani N Kern TS Subauste CS. CD40 promotes the development of early diabetic retinopathy in mice. Diabetologia. (2014) 57:2222–31. 10.1007/s00125-014-3321-x 25015056 PMC4291184

[175] Portillo JA Schwartz I Zarini S Bapputty R Kern TS Gubitosi-Klug RA. Proinflammatory responses induced by CD40 in retinal endothelial and muller cells are inhibited by blocking CD40-Traf2,3 or CD40-Traf6 signaling. Invest Ophthalmol Vis Sci. (2014) 55:8590–7. 10.1167/iovs.14-15340 25477319 PMC4280881

[176] Portillo JC Lopez Corcino Y Dubyak GR Kern TS Matsuyama S Subauste CS. Ligation of CD40 in human muller cells induces P2X7 receptor-dependent death of retinal endothelial cells. Invest Ophthalmol Vis Sci. (2016) 57:6278–86. 10.1167/iovs.16-20301 27893093 PMC5119488

[177] Akira S Takeda K. Toll-like receptor signalling. Nat Rev Immunol. (2004) 4:499–511. 10.1038/nri1391 15229469

[178] El-Asrar AM Nawaz MI Kangave D Geboes K Ola MS Ahmad S Al-Shabrawey M. High-mobility group box-1 and biomarkers of inflammation in the vitreous from patients with proliferative diabetic retinopathy. Mol Vis. (2011) 17:1829–38. 21850157 PMC3137555

[179] Ebihara N Chen L Tokura T Ushio H Iwatsu M Murakami A. Distinct functions between toll-like receptors 3 and 9 in retinal pigment epithelial cells. Ophthalmic Res. (2007) 39:155–63. 10.1159/000103235 17534115

[180] Rajamani U Jialal I. Hyperglycemia induces Toll-like receptor-2 and−4 expression and activity in human microvascular retinal endothelial cells: implications for diabetic retinopathy. J Diabetes Res. (2014) 2014:790902. 10.1155/2014/790902 25610879 PMC4293793

[181] Ko MK Saraswathy S Parikh JG Rao NA. The role of TLR4 activation in photoreceptor mitochondrial oxidative stress. Invest Ophthalmol Vis Sci. (2011) 52:5824–35. 10.1167/iovs.10-6357 21666244 PMC3176080

[182] Samuels IS Portillo JC Miao Y Kern TS Subauste CS. Loss of CD40 attenuates experimental diabetes-induced retinal inflammation but does not protect mice from electroretinogram defects. Vis Neurosci. (2017) 34:E009. 10.1017/S0952523817000074 28965505 PMC8893597

[183] Lee CA Li G Patel MD Petrash JM Benetz BA Veenstra A. Diabetes-induced impairment in visual function in mice: contributions of p38 MAPK, rage, leukocytes, and aldose reductase. Invest Ophthalmol Vis Sci. (2014) 55:2904–10. 10.1167/iovs.13-11659 23920367 PMC4010365

[184] Kovacs B Lumayag S Cowan C Xu S. MicroRNAs in early diabetic retinopathy in streptozotocin-induced diabetic rats. Invest Ophthalmol Vis Sci. (2011) 52:4402–9. 10.1167/iovs.10-6879 21498619

[185] Wu JH Gao Y Ren AJ Zhao SH Zhong M Peng YJ. Altered microRNA expression profiles in retinas with diabetic retinopathy. Ophthalmic Res. (2012) 47:195–201. 10.1159/000331992 22156553

[186] Xiong F Du X Hu J Li T Du S Wu Q. Altered retinal microRNA expression profiles in early diabetic retinopathy: an *in silico* analysis. Curr Eye Res. (2014) 39:720–9. 10.3109/02713683.2013.872280 24502381

[187] Gong Q Su G. Roles of miRNAs and long noncoding RNAs in the progression of diabetic retinopathy. Biosci Rep. (2017) 37. 10.1042/BSR20171157 29074557 PMC5705777

[188] Li Z Dong Y He C Pan X Liu D Yang J. RNA-Seq revealed novel non-proliferative retinopathy specific circulating MiRNAs in T2DM patients. Front Genet. (2019) 10:531. 10.3389/fgene.2019.00531 31275351 PMC6593299

[189] Usui-Ouchi A Ouchi Y Kiyokawa M Sakuma T Ito R Ebihara N. Upregulation of Mir-21 levels in the vitreous humor is associated with development of proliferative vitreoretinal disease. PLoS ONE. (2016) 11:e0158043. 10.1371/journal.pone.0158043 27351379 PMC4924816

[190] Mortuza R Feng B Chakrabarti S. miR-195 regulates SIRT1-mediated changes in diabetic retinopathy. Diabetologia. (2014) 57:1037–46. 10.1007/s00125-014-3197-9 24570140

[191] Chen Q Qiu F Zhou K Matlock HG Takahashi Y Rajala RVS. Pathogenic role of microRNA-21 in diabetic retinopathy through downregulation of PPARalpha. Diabetes. (2017) 66:1671–82. 10.2337/db16-1246 28270521 PMC5440012

[192] Lu JM Zhang ZZ Ma X Fang SF Qin XH. Repression of microRNA-21 inhibits retinal vascular endothelial cell growth and angiogenesis via PTEN dependent-PI3K/Akt/VEGF signaling pathway in diabetic retinopathy. Exp Eye Res. (2020) 190:107886. 10.1016/j.exer.2019.107886 31759996

[193] La Sala L Mrakic-Sposta S Micheloni S Prattichizzo F Ceriello A. Glucose-sensing microRNA-21 disrupts ROS homeostasis and impairs antioxidant responses in cellular glucose variability. Cardiovasc Diabetol. (2018) 17:105. 10.1186/s12933-018-0748-2 30037352 PMC6055345

[194] Fouad M Salem I Elhefnawy K Raafat N Faisal A. MicroRNA-21 as an early marker of nephropathy in patients with type 1 diabetes. Indian J Nephrol. (2020) 30:21–5. 10.4103/ijn.IJN_80_19 32015595 PMC6977383

[195] Qiu F Tong H Wang Y Tao J Wang H Chen L. Inhibition of miR-21-5p suppresses high glucose-induced proliferation and angiogenesis of human retinal microvascular endothelial cells by the regulation of AKT and ERK pathways via maspin. Biosci Biotechnol Biochem. (2018) 82:1366–76. 10.1080/09168451.2018.1459179 29658404

[196] Zhang R Garrett Q Zhou H Wu X Mao Y Cui X. Upregulation of miR-195 accelerates oxidative stress-induced retinal endothelial cell injury by targeting mitofusin 2 in diabetic rats. Mol Cell Endocrinol. (2017) 452:33–43. 10.1016/j.mce.2017.05.009 28487236

[197] Bai Y Bai X Wang Z Zhang X Ruan C Miao J. MicroRNA-126 inhibits ischemia-induced retinal neovascularization via regulating angiogenic growth factors. Exp Mol Pathol. (2011) 91:471–7. 10.1016/j.yexmp.2011.04.016 21586283

[198] Ye P Liu J He F Xu W Yao K. Hypoxia-induced deregulation of miR-126 and its regulative effect on VEGF and MMP-9 expression. Int J Med Sci. (2014) 11:17–23. 10.7150/ijms.7329 24396282 PMC3880987

[199] Rezk NA Sabbah NA Saad MS. Role of MicroRNA 126 in screening, diagnosis, and prognosis of diabetic patients in Egypt. IUBMB Life. (2016) 68:452–8. 10.1002/iub.1502 27118517

[200] Barutta F Bruno G Matullo G Chaturvedi N Grimaldi S Schalkwijk C. MicroRNA-126 and micro-/macrovascular complications of type 1 diabetes in the EURODIAB Prospective Complications Study. Acta Diabetol. (2017) 54:133–. 10.1007/s00592-016-0915-4 27696070

[201] Mazzeo A Beltramo E Iavello A Carpanetto A Porta M. Molecular mechanisms of extracellular vesicle-induced vessel destabilization in diabetic retinopathy. Acta Diabetol. (2015) 52:1113–9. 10.1007/s00592-015-0798-9 26282100

[202] Wang Q Bozack SN Yan Y Boulton ME Grant MB Busik JV. Regulation of retinal inflammation by rhythmic expression of MiR-146a in diabetic retina. Invest Ophthalmol Vis Sci. (2014) 55:3986–94. 10.1167/iovs.13-13076 24867582 PMC4073658

[203] Ye EA Steinle JJ. miR-146a suppresses STAT3/VEGF pathways and reduces apoptosis through IL-6 signaling in primary human retinal microvascular endothelial cells in high glucose conditions. Vision Res. (2017) 139:15–22. 10.1016/j.visres.2017.03.009 28433754 PMC5693785

[204] Feng B Chen S McArthur K Wu Y Sen S Ding Q. miR-146a-Mediated extracellular matrix protein production in chronic diabetes complications. Diabetes. (2011) 60:2975–84. 10.2337/db11-0478 21885871 PMC3198068

[205] Fulzele S El-Sherbini A Ahmad S Sangani R Matragoon S El-Remessy A. MicroRNA-146b-3p regulates retinal inflammation by suppressing adenosine deaminase-2 in diabetes. Biomed Res Int. (2015) 2015:846501. 10.1155/2015/846501 25815338 PMC4359882

[206] McArthur K Feng B Wu Y Chen S Chakrabarti S. MicroRNA-200b regulates vascular endothelial growth factor-mediated alterations in diabetic retinopathy. Diabetes. (2011) 60:1314–23. 10.2337/db10-1557 21357793 PMC3064105

[207] Murray AR Chen Q Takahashi Y Zhou KK Park K Ma JX. MicroRNA-200b downregulates oxidation resistance 1 (Oxr1) expression in the retina of type 1 diabetes model. Invest Ophthalmol Vis Sci. (2013) 54:1689–97. 10.1167/iovs.12-10921 23404117 PMC3626515

[208] Malik AN Parsade CK Ajaz S Crosby-Nwaobi R Gnudi L Czajka A Sivaprasad S. Altered circulating mitochondrial DNA and increased inflammation in patients with diabetic retinopathy. Diabetes Res Clin Pract. (2015) 110:257–65. 10.1016/j.diabres.2015.10.006 26625720

[209] Loukovaara S Piippo N Kinnunen K Hytti M Kaarniranta K Kauppinen A. NLRP3 inflammasome activation is associated with proliferative diabetic retinopathy. Acta Ophthalmol. (2017) 95:803–8. 10.1111/aos.13427 28271611

[210] Thounaojam MC Montemari A Powell FL Malla P Gutsaeva DR Bachettoni A. Monosodium urate contributes to retinal inflammation and progression of diabetic retinopathy. Diabetes. (2019) 68:1014–25. 10.2337/db18-0912 30728185 PMC6477903

[211] Shalaby L Thounaojam M Tawfik A Li J Hussein K Jahng WJ. Role of endothelial ADAM17 in early vascular changes associated with diabetic retinopathy. J Clin Med. (2020) 9:400. 10.3390/jcm9020400 32024241 PMC7073770

[212] Du MR Yan L Li NS Wang YJ Zhou T Jiang JL. Asymmetric dimethylarginine contributes to retinal neovascularization of diabetic retinopathy through EphrinB2 pathway. Vascul Pharmacol. (2018) 108:46–56. 10.1016/j.vph.2018.05.004 29777874

